# Activation of Thioglycosides with Ferric Chloride: Scope and Mechanism

**DOI:** 10.1021/acsomega.6c03149

**Published:** 2026-05-21

**Authors:** Lacie M. Ridgway, Rachel L. Turecki, Faranak Pooladian, Anupama Das, Alexei V. Demchenko

**Affiliations:** Department of Chemistry, 7547 Saint Louis University, 3501 Laclede Avenue, St. Louis, Missouri 63103, United States

## Abstract

Previously, we disclosed a new method for the activation of armed and superarmed ethylthio glycosides using an inexpensive and abundant iron­(III) chloride. Reported herein are the next steps toward refining the reaction conditions, broadening the scope of this method to glycosidation of less-reactive glycosyl donors, and investigating the reaction mechanism. This reaction is swift and generally high-yielding. The completion of the reaction can be monitored by eye, and typically, the color change (start of the reaction) can be detected right after the addition of the glycosyl donor to the premixed mixture of the acceptor and ferric chloride.

## Graphical abstract

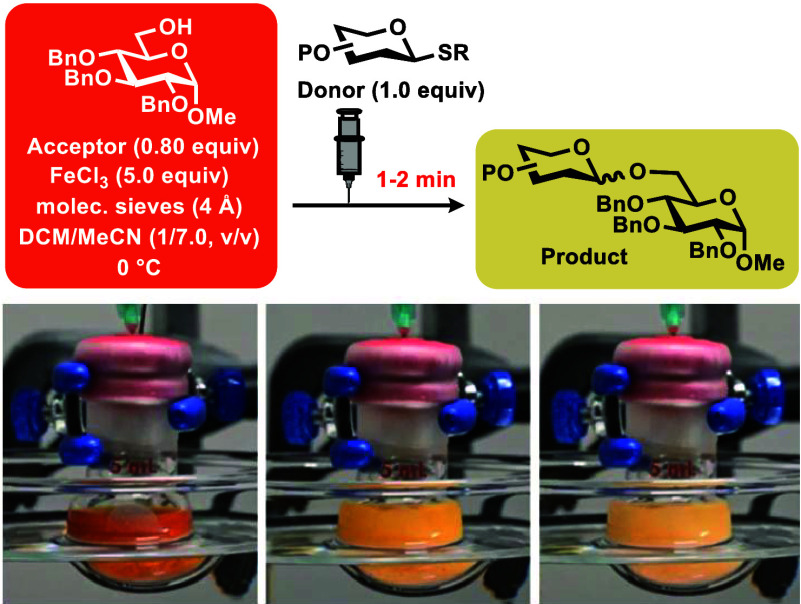

## Introduction

Carbohydrates are both the most abundant organic compounds in nature and popular targets for pharmaceutical development. The development of effective methods for synthesizing carbohydrates to improve understanding of their functions and boost their practical applications is an important direction in modern science. While synthetic strategies for glycosylation have been thoroughly investigated, the development of greener methods and less-toxic reagents is imperative to further advancing glycochemistry. Thioglycosides are commonly used building blocks due to their ability to tolerate a majority of reaction conditions associated with protecting group installation and removal.^1–4^ Thioglycosides are also popular glycosyl donors because their activation for glycosylation can be achieved with relatively mild electrophilic promoters.^4^ Besides common systems comprising organosulfur reagents,^5–10^ halogens,^11–16^ or photoactivators,^17–20^ studying promoters based on salts of transition metals has emerged as a promising avenue for further exploration.^21^ Since early reports by Ferrier,^22^ Van Cleve,^23^ Hanessian,^24^ and Garegg,^25^ which showcased that thioglycosides (**A**, Figure 1) and thioimidates (**B**, Hanessian) can be activated with mercury­(II) salts, an immense progress has been made. Copper­(II),^26–28^ silver­(I),^29–33^ and bismuth­(III)^34^ salts have been investigated with a variety of thioimidates (such as *S*-benzoxazolyl, **C**). Copper­(II) was later on found to be effective for the activation of alkylthio and arylthio glycosides.^35,36^ Pohl and co-workers discovered that Ph_3_Bi­(OTf)_2_ can also activate thioglycosides.^37^ A detailed mechanistic study revealed that anomerization of the initial β-*S*-propyl donor into its α-linked anomer is required for glycosylation to occur (**D**).^38,39^ Kartha reported the use of In­(OTf)_3_ as a single promoter for the activation of thioglycosides.^40^ Their study suggested that the anomeric sulfur atom complexes with the metal center in In­(OTf)_3_, allowing for the formation of an activated intermediate (**E**). Sureshan^41^ and Zhu^42^ described the activation of a variety of thioglycosides using AuCl_3_ or AuBr_3_ without any copromoter. Their studies indicated that the activation was also achieved through direct coordination of the metal center with the anomeric sulfur (**F**). The activation of conventional thioglycosides with PdBr_2_ was reported by our group.^43^ It was found that the activation can be performed in the presence of PdBr_2_ alone, but the presence of an additive (propargyl bromide) accelerates the activation process. A preliminary mechanistic analysis relying on ^1^H NMR spectroscopy revealed that propargyl bromide additive could form a more reactive reaction intermediate (**G**).

**Figure 1:**
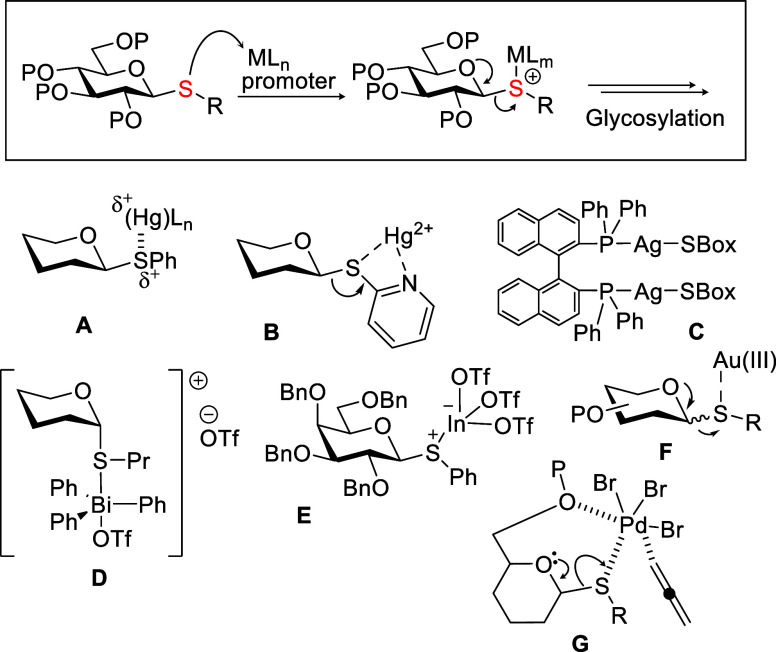
Activation of thioglycosides with transition metals.

While the previous studies were quite successful, the need to identify greener, cheaper, and more accessible transition-metal salts capable of activating thioglycosides emerged as a timely avenue for further expansion. Previously, we described a new protocol for the activation of ethylthio glycosides in the presence of ferric chloride without any copromoters or additives.^44^ A series of highly reactive (armed and superarmed) glycosyl donors were glycosidated with both primary and secondary acceptors in good yields. The reactions were somewhat sluggish, which prevented the use of the developed reaction conditions in application to less-reactive (disarmed or superdisarmed) glycosyl donors.

Following initial observations of the effect of protecting groups on reactivity,^45^ Fraser-Reid was the first to rationalize activating (arming) and deactivating (disarming) effects in glycosylation.^46,47^ Thus, it was observed that electronically activated, benzylated (armed) glycosyl donors are more reactive than their electronically deactivated, acylated counterparts. Extended studies of this phenomenon performed by Fraser-Reid,^48^ Ley,^49,50^ and Wong^51,52^ revealed several glycosyl donors that were outside the scope of the armed-disarmed boundary. This discovery opened a new research direction,^53^ and our group reported that 2-*O*-benzyl-3,4,6-tri-*O*-benzoyl-protected glycosyl donors are even less reactive (superdisarmed) than their disarmed per-*O*-benzoylated counterparts.^54^ This surprising reactivity was rationalized by the existence of the O-2/O-5 cooperative effect, and the enhanced understanding of glycosyl donor reactivity led to the discovery of 2-*O*-benzyl-3,4,6-tri-*O*-benzoyl protected donors that were more reactive (superarmed) than armed per-*O*-benzylated glycosyl donors.^55–57^ Other modes to superdisarm^58^ and superarm^59,60^ glycosyl donors have also been explored.^53,61–63^

Herein, we report a new protocol for efficient and versatile glycosidation of an expanded range of thioglycosides equipped with different leaving and protecting groups in the presence of iron­(III) chloride. Also reported are the preliminary mechanistic studies.

## Results and Discussion

One previous result that sparked our attention and prompted this investigation was the reaction of benzoylated galactosyl donor **1** with glycosyl acceptor **2**. This was the only successful attempt to activate the disarmed donor because in all other attempts, the yields were below the practical preparative range. In this case, disaccharide **3** was obtained in 1 h in a moderate yield of 77% (entry 1, Table 1). The reaction conditions comprised FeCl_3_ (5.0 equiv) and 3 Å molecular sieves in DCM/MeCN (1/1.0, v/v) at 0 °C. In comparison, all armed and superarmed glucosyl and galactosyl donors (**4**, **6**, **8**, and **10**) produced the respective disaccharides (**5**, **7**, **9**, and **11**) in 1 h in excellent yields of 87–96% (previous results are surveyed in entries 2–5). While the lower yield of disaccharide **3** derived from disarmed glycosyl donor **1** was not unexpected, the surprise was that along with the expected disaccharide **3** also formed and isolated was tetra-*O*-benzoylated glycosyl chloride. We note that our attempts to use these reaction conditions to perform the synthesis of glycosyl chlorides were deemed unreliable and poorly reproducible. In addition, these reactions led to substantial quantities of the hemiacetal byproduct, which was formed as a result of a competing hydrolysis reaction.

**Table 1:**
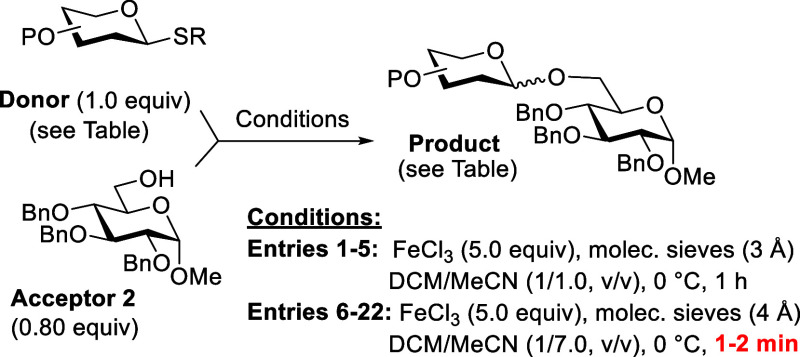

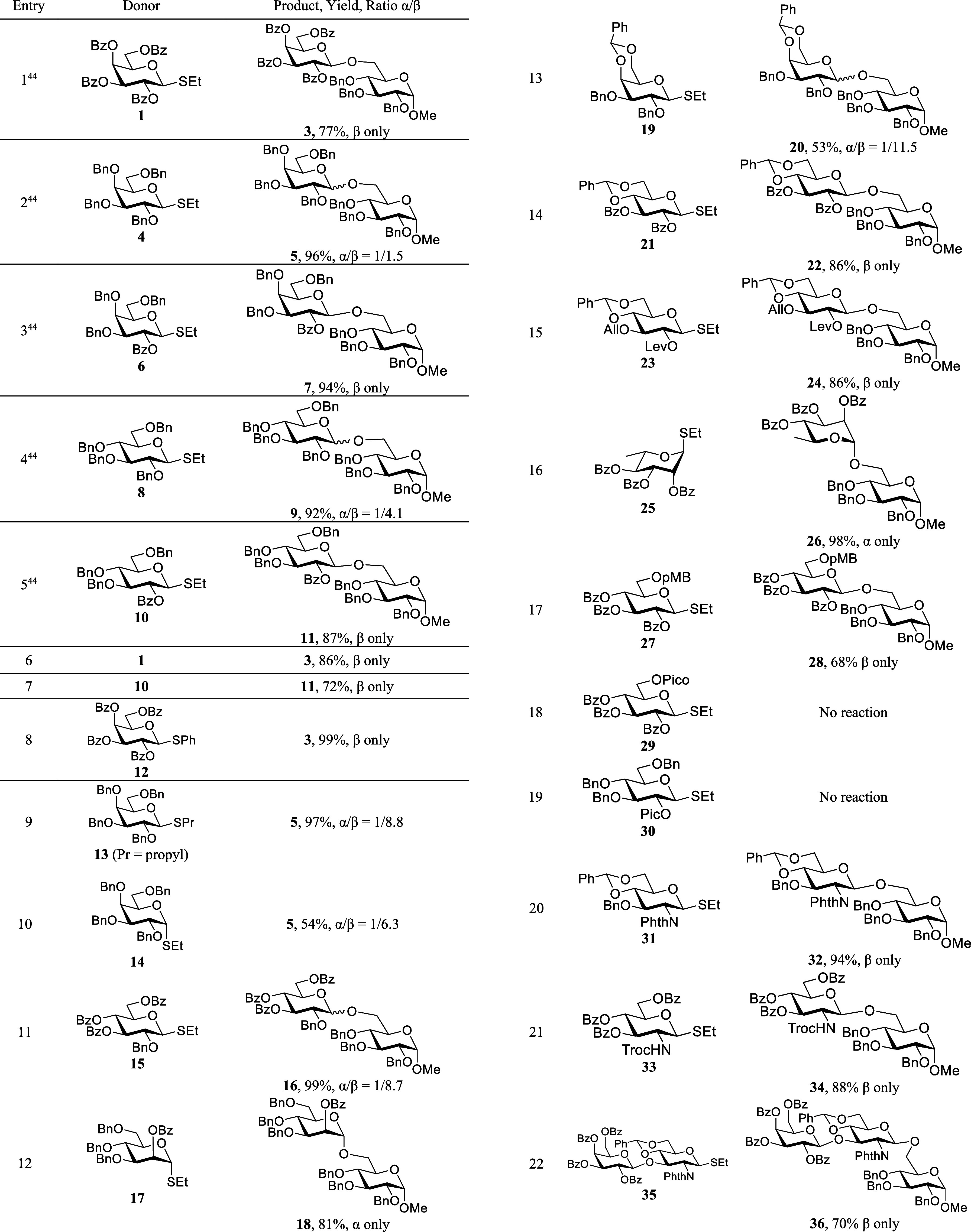
Investigation of the Scope of Glycosyl Donors

The discovery of glycosyl chloride among the products of the reaction led us to the first question. Does the ferric chloride-promoted glycosylation proceed through the glycosyl chloride intermediate? Our group had previously reported that the activation of glycosyl chlorides can be affected in the presence of catalytic (20 mol %) amounts of ferric chloride.^64^ However, applying our reaction conditions (5.0 equiv of FeCl_3_ in DCM/MeCN) to tetra-*O*-benzoylated galactosyl chloride and then injecting anhydrous methanol showed no formation of the respective methyl glycoside, even after 24 h. This result answered the first question: chloride is not a reactive intermediate en route to glycosylation but rather a byproduct formed under these reaction conditions. However, this result also raises another question. What is the actual promoter in this reaction if the reaction conditions are incapable of activating glycosyl chlorides? Apparently, it is not FeCl_3_, which would have readily activated the glycosyl chloride.

Hence, we endeavored to further investigate this reaction. The use of DCM alone gave low yields, and the use of MeCN as a coordinating cosolvent seemed necessary for this study. On the one hand, ferric chloride is insoluble in DCM, and on the other hand, it has been reported that phosgene could form when a solution of ferric chloride in DCM was exposed to visible light.^65^ Thus, the application of DCM as the sole solvent for these reactions was deemed impractical and was ceased due to safety concerns. Green solvents capable of coordination to ferric chloride, such as acetone, 2-methyltetrahydrofuran (2-MTHF), dimethyl sulfoxide (DMSO), or dimethyl isosorbide (DMI) were practically ineffective, and in the majority of attempts, only poor yields of disaccharides were recorded (from traces to 15%, results are not shown). In our previous study, DCM/MeCN (1/1.0, v/v) was employed, and investigation of glycosylation using these solvents in various ratios has continued. To our delight, when we increased the proportion of MeCN to DCM/MeCN (1/7.0, v/v) and replaced 3 Å molecular sieves with 4 Å, we observed very swift reactions and excellent yields. By using these reaction conditions, each glycosylation was completed within 1–2 min.

We also began understanding the mechanistic aspects of the reaction. The complexation of ferric chloride to MeCN has been well-studied. Upon solvation in MeCN, ferric chloride forms a complex with acetonitrile with concomitant dissociation of a chloride, which gets picked by another molecule of ferric chloride. This results in the formation of a blood-red-colored tetrakis­(acetonitrile)­dichloro iron­(III) cation and tetrachloroferrate anion.^66,67^ Since the reaction before the addition of the donor is of blood-red color, we postulate that the active promoter of the glycosylation reaction is [FeCl_2_(MeCN)_4_] [FeCl_4_] depicted in Figure 2A. The blood-red color of the initial solution rapidly (in 1–2 min) changes to the beige-yellow color that signifies completion of the reaction. The color change could be attributed to the change in the coordination environment, such as chelation to the leaving group and reduction of iron­(III) to iron­(II). The color change of the initial solution containing glycosyl acceptor, [FeCl_2_(MeCN)_4_]­[FeCl_4_] complex, and molecular sieves starts practically instantaneously upon injection of the donor (see Figure 2B and the supplied real-time video of the reaction as a part of the Supporting Information). This allows for naked-eye reaction monitoring, if so desired.^68^

**Figure 2:**
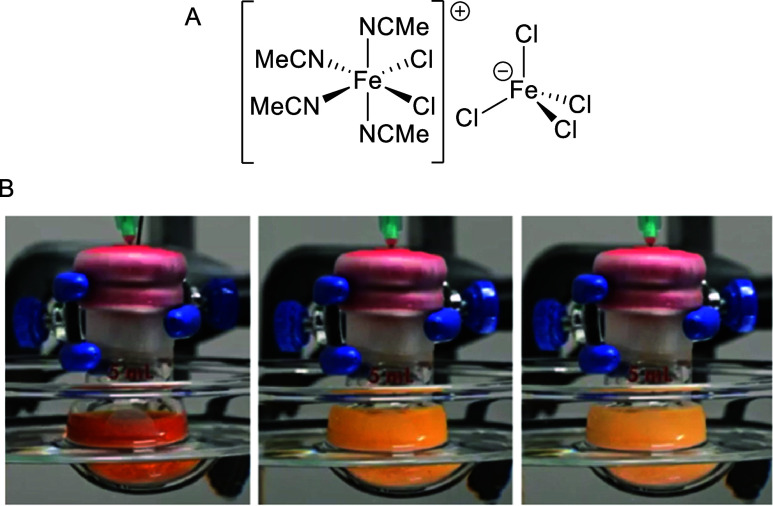
Tetrakis­(acetonitrile)­dichloro iron­(III) tetrachloroferrate (A) and naked-eye reaction monitoring (B).

With the most favorable reaction conditions established as follows: FeCl_3_ (5.0 equiv), 4 Å molecular sieves, DCM/MeCN (1/7.0, v/v), at 0 °C, we continued investigating glycosylation to expand its scope. A variety of glycosyl donors were glycosidated with the primary glycosyl acceptor **2**. These studies are surveyed in Table 1. All reactions were completed within 1–2 min, and the completion of the reaction was monitored by eye. We first endeavored to repeat some of our previous glycosylation reactions using the new reaction conditions. Glycosidation of benzoylated galactosyl donor **1** with glycosyl acceptor **2** proceeded smoothly, and disaccharide **3** was obtained in an improved yield of 86% yield (entry 6). Glycosidation of the superarmed glycosyl donor **10** with glycosyl acceptor **2** proceeded swiftly, and disaccharide **11** was obtained in 72% yield (entry 7).

We attribute this decreased yield to the fact that the new reaction conditions are too powerful for controlling the rate of glycosidation of highly reactive glycosyl donors and might be more applicable to glycosidation of less-reactive substrates. In fact, this reaction was accompanied by the production of other unidentified compounds, which complicated the isolation of the pure product and also contributed to the decreased yield. In stark contrast, glycosidation of phenylthio glycosyl donor **12** equipped with the disarming protecting group pattern, 2,3,4,6-tetra-*O*-benzoyl, produced disaccharide **3** in an excellent yield of 99% with complete β-selectivity (entry 8). Armed per-*O-*benzylated galactosyl donor **13** bearing the propylthio leaving group led to disaccharide **5** in an excellent yield of 97% (α/β = 1/8.8, entry 9). We note that although no neighboring group participation was possible, we still obtained very good β-selectivity. This is likely attributed to the solvent effect, as MeCN is known to be a β-directing participating solvent. Upon glycosidation of armed α-galactosyl donor **14**, we obtained disaccharide **5** in 54% yield (α/β = 1/6.3, entry 10). We attribute this moderate yield to enhanced stability of α-thioglycosides in general, and this reaction may need subsequent investigation. The application of superdisarmed 2-*O*-benzyl-3,4,6-tri-*O*-benzoyl-protected donor **15** led to disaccharide **16** in 99% yield (α/β = 1/8.7, entry 11). The glycosidation of mannosyl donor **17** equipped with the superarming protecting group pattern, 2-*O*-benzoyl-3,4,6-tri-*O*-benzyl, produced disaccharide **18** in 81% yield (α only, entry 12).

To evaluate the use of ferric chloride on substrates equipped with labile protecting groups, we began with a series of glycosyl donors equipped with acid-labile 4,6-*O*-benzylidene acetal protecting groups. 2,3-Di-*O*-benzylated galactosyl donor **19** afforded disaccharide **20** in a moderate yield of 53%, albeit with excellent β-selectivity (α/β = 1/11.5, entry 13). Differently protected 4,6-*O*-benzylidene glucosyl donors **21** and **23** produced the respective disaccharides **22** and **24** in identical 86% yield (β only, entries 14 and 15). Upon the use of the disarmed rhamnose donor **25**, disaccharide **26** was obtained in 98% yield (α only, entry 16).

Upon the use of substrates containing protecting groups previously reported to be labile in the presence of ferric chloride,^69,70^ disarmed glucosyl donor **27** equipped with the *p*-methoxybenzyl protecting group at the primary position, produced disaccharide **28** in 68% yield (β only, entry 17). Also isolated was the 6-*O*-*p*-methoxybenzylated acceptor (23%). Glycosyl donor **29** protected with the picoloyl group at the primary position could not be glycosidated under these reaction conditions (entry 18). Ferric chloride removes picoloyl, but herein we did not notice any deprotection. The inability to activate and glycosidate this donor was attributed to chelation of the ferric center with the nitrogen atom of the picoloyl group. Similarly, glycosyl donor **30** equipped with 2-*O-*picolinyl group could not be glycosidated under these reaction conditions (entry 19).

Subsequently, we investigated the use of ferric chloride for the activation of aminosugar donors. Glucosamine donors **31** and **33** bearing the *N*-phthalimido and *N*-2,2,2-trichloroethoxycarbonyl (Troc) protecting groups, respectively, smoothly produced disaccharides **32** and **34** in excellent yields of 88–94% with complete β-selectivity in both cases (entries 20–21). Also, disaccharide donor **35** bearing the *N*-phthalimido protecting group smoothly produced disaccharide **36** in 70% yield with complete β-selectivity (entry 22).

We then turned our attention to investigating other glycosyl acceptors using glycosyl donor **13** under standard reaction conditions. Glycosylation of secondary glycosyl acceptors **37** and **39** was very efficient, and the respective disaccharides **38** and **40** were obtained in 90–92% yield (entries 1 and 2, Table 2). Glycosylation of hindered 4-OH acceptor **41** was less efficient; nevertheless, disaccharide **42** was obtained in a respectable yield of 81% (entry 3). Finally, glycosylation of electronically deactivated 6-OH acceptor **43** produced disaccharide **44** in 89% yield (entry 4). These reactions were less stereoselective (α/β = 1/1.5–2.0) in comparison to those with the benzylated primary acceptor **2**. However, we do not yet have an explanation for this decreased stereoselectivity.

**Table 2:**
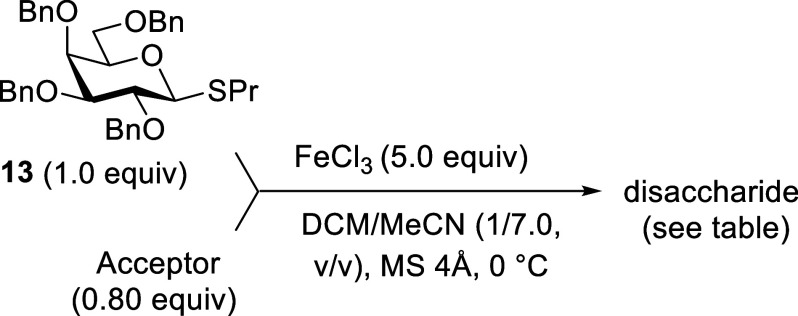

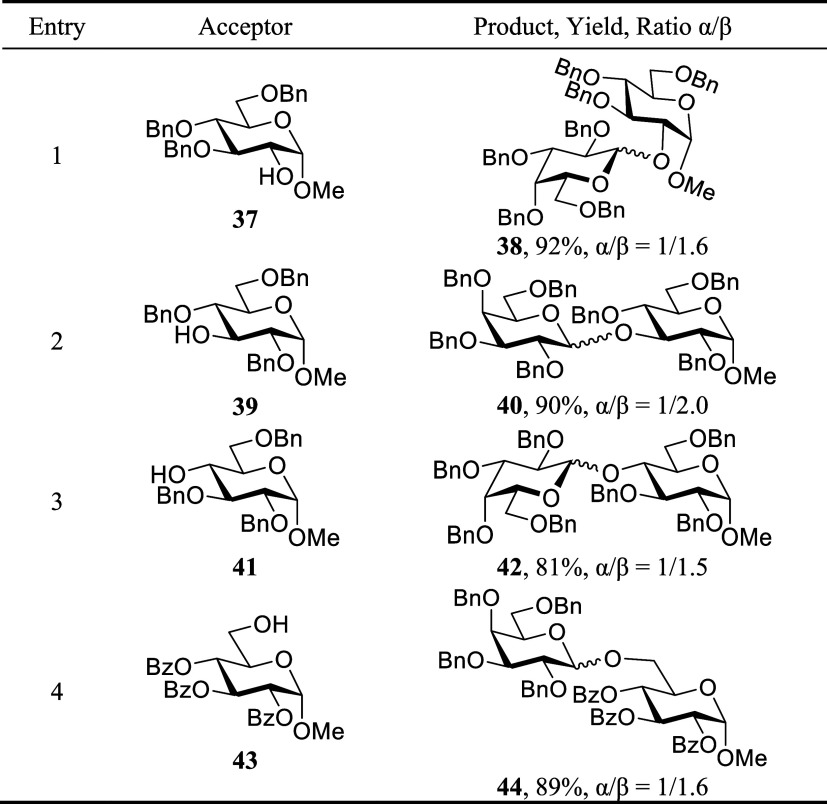
Broadening the Scope of the FeCl_3_-Assisted Glycosylation to Other Glycosyl Acceptors

Having demonstrated very effective and swift ferric chloride-promoted glycosidations of thioglycoside donors with both primary and secondary acceptors, we endeavored to determine a plausible reaction mechanism. Due to the paramagnetic nature of iron­(III), mechanistic studies of this glycosylation were met with great difficulty due to the inability to utilize NMR spectroscopy. Determination of the mechanistic pathway, therefore, required more in-depth analysis. Plausible mechanisms for ferric chloride-promoted reactions have been reported. Naturally abundant, inexpensive, and relatively benign,^71^ ferric chloride has been applied both to the protecting groups manipulations^72,73^ and to the glycosidation of other classes of glycosyl donors, including acetate,^74–83^ aryl glycoside,^84^ pivaloate,^85^ bromide,^86^ various imidates,^87,88^ chloride,^64^ or propargyl glycoside.^89^ Thus, Gosh and co-workers proposed the activation of glycosyl trichloroacetimidates through chelation of the ferric center to the imidate nitrogen and C-2-oxygen of the donor, producing intermediate **A** leading to complete β-selectivity of subsequent glycosylation (Figure 3).^87^ Yu and co-workers proposed that the complexation of stoichiometric ferric chloride (1.0 equiv to glycosyl acceptor) in acetonitrile produced a ferric complex incapable of activating the glycosyl *N*-aryltrifluoroacetimidate donor.^88^ However, the ferric complex coordinated to the hydroxyl group of the acceptor (**B**, Figure 3) could lead to protonation of the imidate nitrogen, thus activating the glycosyl donor. Sun et al. proposed a mechanism of activation for propargyl glycoside donors in which activation occurs due to π-complexation of the ferric center to the alkyne and the anomeric oxygen (**C**).^89^

**Figure 3:**
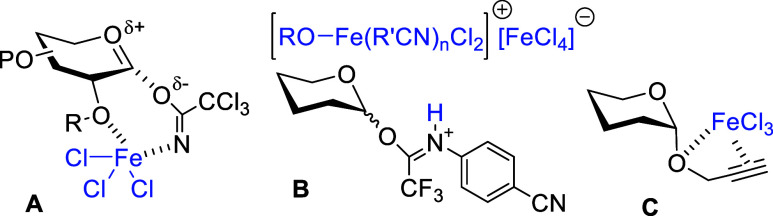
Proposed mechanisms for ferric chloride-promoted glycosylation of trichloroacetimidate (A), glycosyl *N*-aryltrifluoroacetimidate (B), and propargyl glycoside (C) donors.

Our reaction seems to require 5.0 equiv of ferric chloride. On the basis of our observations with differentially substituted glycosyl donors of various sugar series surveyed in tables, we hypothesize that the activation occurs by chelation of both the glycosyl donor and the glycosyl acceptor (Scheme 1). 2.0 equiv of ferric chloride can generate 1.0 equiv of promoter [FeCl_2_(MeCN)_4_] [FeCl_4_]. When 2.0 equiv of ferric chloride was added in the absence of the glycosyl acceptor, the complexation of the donor was observed by the appearance of a prominent baseline spot on the TLC. However, the leaving group fails to depart when only 2.0 equiv of ferric chloride is used. This led us to a hypothesis that another equivalent of [FeCl_2_(MeCN)_4_] [FeCl_4_] is needed to coordinate the glycosyl acceptor. This dual action seems to be necessary for successful glycosylation to take place.

**Scheme 1:**
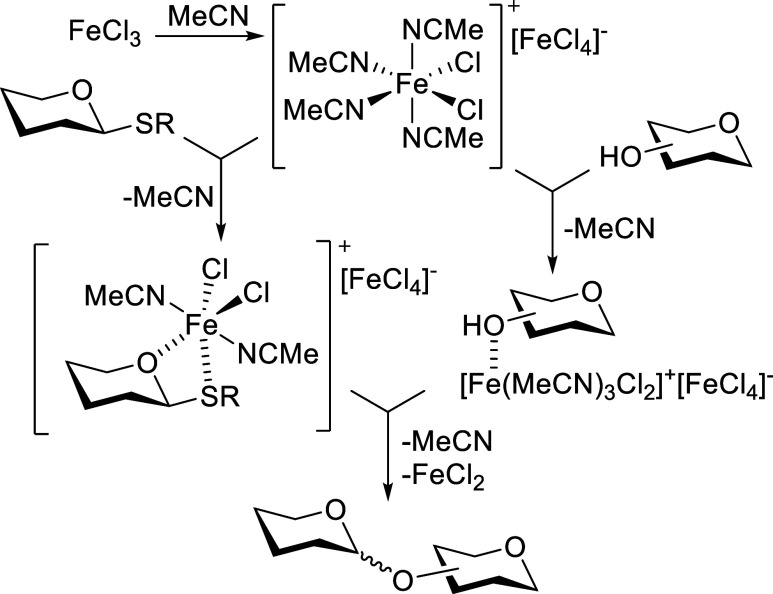
Plausible Reaction Mechanism for Ferric Chloride-Promoted Glycosidation of Thioglycosides

## Conclusions

Upon discovery of glycosyl chloride formation, found during purification of disaccharide **3**, further optimization of reaction conditions determined that chlorination of glycosides is possible using ferric chloride; however, substantial competing hydrolysis rendered the reaction inefficient and poorly reproducible. Although studies of chlorination were discontinued, it was determined that the newly optimized preactivated conditions could efficiently activate the glycosyl donor, leading to swift and high-yielding reactions that could be monitored by the naked eye. An expanded substrate scope was performed, in which donors bearing a variety of protecting groups were glycosylated to the primary and secondary glycosyl acceptors, producing disaccharides in very good to excellent yields. Lower yields were obtained with highly reactive, superarmed glycosyl donors because these reactions were accompanied by the formation of side products due to the reactivity mismatch. A mechanism for the glycosidation of thioglycosides using FeCl_3_ was proposed, according to which the ferric chloride complex coordinates both the leaving group of the donor and the hydroxyl group of the acceptor.

## Experimental Section

### General

All chemicals used were reagent grade and used as supplied. The ACS grade solvents used were purified and dried in accordance with standard procedures. Column chromatography was performed on silica gel 60 (230–400 mesh). Reactions were monitored by TLC on Kieselgel 60 F254. Compounds were detected by examination under UV light and by subsequent charring with 10% sulfuric acid in methanol. Solvents were removed under reduced pressure at ≤ 40 °C. CH_2_Cl_2_ was distilled from CaH_2_ directly prior to application. Molecular sieves (4 Å) were crushed and activated *in vacuo* at 390 °C for 8 h in the first instance and then for 3 h at 390 °C directly prior to application. ^1^H NMR spectra were recorded at 400 MHz, ^13^C NMR spectra were recorded at 101 MHz. The ^1^H NMR chemical shifts are referenced to tetramethylsilane (0 ppm) or CHCl_3_ (7.26 ppm) for ^1^H NMR spectra for solutions in CDCl_3_. Anomeric ratios (if applicable) were determined by comparison of the integral intensities of relevant signals in ^1^H NMR spectra (see the Supporting Information). Accurate mass spectrometry determinations were performed using Agilent 6230 ESI TOF LCMS mass spectrometer.

### Synthesis of Building Blocks

#### Ethyl 2,3,4,6-Tetra-*O*-benzoyl-1-thio-β-d-galactopyranoside (**1**)

**1** was synthesized as reported previously, and its analytical data were in accordance with that previously described.^90^

#### Methyl 2,3,4-Tri-*O*-benzyl-α-d-glucopyranoside (**2**)

**2** was synthesized as reported previously, and its analytical data were in accordance with that previously described.^91^

#### Ethyl 2,3,4,6-Tetra-*O*-benzyl-1-thio-β-d-galactopyranoside (**4**)

**4** was synthesized as reported previously, and its analytical data were in accordance with those previously described.^13,92^

#### Ethyl 2-*O*-Benzoyl-3,4,6-tri-*O*-benzyl-1-thio-β-d-galactopyranoside (**6**)

**6** was synthesized as reported previously, and its analytical data were in accordance with those previously described.^93,94^

#### Ethyl 2,3,4,6-Tetra-*O*-benzyl-1-thio-β-d-glucopyranoside (**8**)

**8** was synthesized as reported previously, and its analytical data were in accordance with that previously described.^95^

#### Ethyl 2-*O*-Benzoyl-3,4,6-tri-*O*-benzyl-1-thio-β-d-glucopyranoside (**10**)

**10** was synthesized as reported previously, and its analytical data were in accordance with those previously described.^94,96^

#### Phenyl 2,3,4,6-Tetra-*O*-benzoyl-1-thio-β-d-galactopyranoside (**12**)

**12** was synthesized as reported previously, and its analytical data were in accordance with that previously described.^97^

#### Propyl 2,3,4,6-Tetra-*O*-benzyl-1-thio-β-d-galactopyranoside (**13**)

**13** was synthesized as reported previously, and its analytical data were in accordance with that previously described.^38^

#### Ethyl 2,3,4,6-Tetra-*O*-benzyl-1-thio-α-d-galactopyranoside (**14**)

**14** was synthesized as reported previously, and its analytical data were in accordance with that previously described.^98^

#### Ethyl 3,4,6-Tri-*O*-benzoyl-2-*O*-benzyl-1-thio-β-d-glucopyranoside (**15**)

**15** was synthesized as reported previously, and its analytical data were in accordance with that previously described.^99^

#### Ethyl 2-*O*-Benzoyl-3,4,6-tri-*O*-benzyl-1-thio-α-d-mannopyranoside (**17**)

**17** was synthesized as reported previously, and its analytical data were in accordance with that previously described.^100^

#### Ethyl 4,6-*O*-Benzylidene-2,3-di-*O*-benzyl-1-thio-β-d-galactopyranoside (**19**)

**19** was synthesized as reported previously, and its analytical data were in accordance with that previously described.^101^

#### Ethyl 2,3-Di-*O*-benzoyl-4,6-*O*-benzylidene-1-thio-β-d-glucopyranoside (**21**)

**21** was synthesized as reported previously, and its analytical data were in accordance with that previously described.^102^

#### Ethyl 3-*O*-Allyl-4,6-*O*-benzylidene-2-*O*-levulinoyl-1-thio-β-d-glucopyranoside (**23**)

**23** was synthesized as reported previously, and its analytical data were in accordance with that previously described.^103^

#### Ethyl 2,3,4-Tri-*O*-benzoyl-1-thio-α-l-rhamnopyranoside (**25**)

**25** was synthesized as reported previously, and its analytical data were in accordance with that previously described.^104^

#### Ethyl 2,3,4-Tri-*O*-benzoyl-6-*O*-*p*-methoxybenzyl-1-thio-β-d-glucopyranoside (**27**)

**27** was synthesized as reported previously, and its analytical data were in accordance with that previously described.^105^

#### Ethyl 2,3,4-Tri-*O*-benzoyl-6-*O*-picoloyl-1-thio-β-d-glucopyranoside (**29**)

**29** was synthesized as reported previously, and its analytical data were in accordance with that previously described.^70^

#### Ethyl 3,4,6-Tri*-O*-benzyl-2-*O*-picolinyl-1-thio-β-d-glucopyranoside (**30**)

**30** was synthesized as reported previously, and its analytical data were in accordance with that previously described.^106^

#### Ethyl 3-*O*-Benzyl-4,6-*O*-benzylidene-2-deoxy-2-phthalimido-1-thio-β-d-glucopyranoside (**31**)

**31** was synthesized as reported previously, and its analytical data were in accordance with that previously described.^107^

#### Ethyl 3,4,6-Tri-*O*-benzoyl-2-deoxy-1-thio-2-(2,2,2-trichloroethoxy)­carbamoyl-β-d-glucopyranoside (**33**)

**33** was synthesized as reported previously, and its analytical data were in accordance with that previously described.^108^

#### Ethyl *O*-(2,3,4,6-Tetra-*O-*benzoyl-β-d-galactopyranosyl)-(1→3)-4,6-*O*-benzylidene-2-deoxy-2-phthalimido-1-thio-β-d-glucopyranoside (**35**)

**35** was synthesized as reported previously, and its analytical data were in accordance with that previously described.^109^

#### Methyl 3,4,6-Tri-*O*-benzyl-α-d-glucopyranoside (**37**)

**37** was synthesized as reported previously, and its analytical data were in accordance with those previously described.^91^

#### Methyl 2,4,6-Tri-*O*-benzyl-α-d-glucopyranoside (**39**)

**39** was synthesized as reported previously, and its analytical data were in accordance with those previously described.^91,110^

#### Methyl 2,3,6-Tri-*O*-benzyl-α-d-glucopyranoside (**41**)

**41** was synthesized as reported previously and its analytical data were in accordance with that previously described.^91^

#### Methyl 2,3,4-Tri-*O*-benzoyl-α-d-glucopyranoside (**43**)

**43** was synthesized as reported previously, and its analytical data were in accordance with those previously described.^111^

### Synthesis of Disaccharides and Trisaccharide **36**

#### General Procedure for Glycosylation

A mixture containing ferric chloride (FeCl_3_, 5.0 equiv to glycosyl donor, 0.220–0.333 mmol) and freshly activated molecular sieves (4 Å, 180 mg) in dry acetonitrile (MeCN, 2.0 mL) was stirred under argon for 2 h at 0 °C. A solution of thioglycoside donor (30 mg, 0.043–0.067 mmol) and glycosyl acceptor (0.034–0.053 mmol) in dry DCM/MeCN (1.2 mL, 1/7.0, v/v) was then injected via syringe and the resulting mixture was stirred under argon for 1–2 min at 0 °C. After that, sat. aq. NaHCO_3_ (0.5 mL) was added and the resulting mixture was stirred for 10 min at rt. The solids were filtered off through a pad of Celite containing anhydrous Na_2_SO_4_ and rinsed successively with DCM. The combined filtrate (∼20 mL) was concentrated under reduced pressure. The residue was purified by column chromatography on silica gel (ethyl acetate–hexane gradient elution or acetone-toluene gradient elution) to afford a disaccharide (or a trisaccharide) derivative in yields and anomeric ratios listed in tables and below.

#### Methyl 6-*O*-(2,3,4,6-Tetra-*O*-benzoyl-β-d-galactopyranosyl)-2,3,4-tri-*O*-benzyl-α-d-glucopyranoside (**3**)

**3** was obtained from thioglycoside **1** and glycosyl acceptor **2** by the general glycosylation method in 86% yield (β only) as a colorless syrup. Compound **3** was also obtained from thioglycoside **12** and glycosyl acceptor **2** by the general glycosylation method in 99% yield (β only) as a colorless syrup. Analytical data for **3** were in accordance with that reported previously.^112^

#### Methyl 2,3,4-Tri-*O*-benzyl-6-*O*-(2,3,4,6-tetra-*O*-benzyl-d-galactopyranosyl)-α-d-glucopyranoside (**5**)

**5** was obtained from thioglycoside **13** and glycosyl acceptor **2** by the general glycosylation method in 97% yield (α/β = 1/8.8) as a colorless syrup. Compound **5** was also obtained from donor **14** in 54% yield (α/β = 1/6.3) Analytical data for **5** were in accordance with that reported previously.^113^

#### Methyl 2,3,4-Tri-*O*-benzyl-6-*O*-(2-*O*-benzoyl-3,4,6-tetra-*O*-benzyl-d-glucopyranosyl)-α-d-glucopyranoside (**11**)

**11** was obtained from thioglycoside **10** and glycosyl acceptor **2** by the general glycosylation method in 72% yield (β only) as a colorless syrup. Analytical data for **5** were in accordance with that reported previously.^113^

#### Methyl 2,3,4-Tri-*O*-benzyl-6-*O*-(3,4,6-tri-*O*-benzoyl-2-*O*-benzyl-d-glucopyranosyl)-α-d-glucopyranoside (**16**)

**16** was obtained from thioglycoside **15** and glycosyl acceptor **2** by the general glycosylation method in 99% yield (α/β = 1/8.7) as a colorless syrup. Analytical data for **16** were in accordance with that reported previously.^99^

#### Methyl 2,3,4-Tri-*O*-benzyl-6-*O*-(2-O-benzoyl-3,4,6-tri-*O*-benzyl-α-d-mannopyranosyl)-α-d-glucopyranoside (**18**)

**18** was obtained from thioglycoside **17** and glycosyl acceptor **2** by the general glycosylation method in 81% yield (α only) as a colorless syrup. Analytical data for **18** were in accordance with that reported previously.^114^

#### Methyl 2,3,4-Tri-*O*-benzyl-6-*O*-(2,3-*O*-benzyl-4,6-*O*-benzylidene-α/β-d-galactopyranosyl)-α-d-glucopyranoside (**20**)

**20** was obtained from thioglycoside **19** and glycosyl acceptor **2** by the general glycosylation method in 53% yield (α/β = 1/11.5) as a white amorphous solid. Analytical data for **20** were in accordance with that reported previously.^115^

#### Methyl 2,3,4-Tri-*O*-benzyl-6-*O*-(2,3-*O*-benzoyl-4,6-*O*-benzylidene-β-d-glucopyranosyl)-α-d-glucopyranoside (**22**)

**22** was obtained from thioglycoside **21** and glycosyl acceptor **2** by the general glycosylation method in 86% yield (β only) as a white amorphous solid. Analytical data for **22** were in accordance with that reported previously.^116^

#### Methyl 2,3,4-Tri-*O*-benzyl-6-*O*-(3-*O*-allyl-4,6-*O*-benzylidene-2-*O*-levulinoyl-β-d-glucopyranosyl)-α-d-glucopyranoside (**24**)

**24** was obtained from thioglycoside **23** and glycosyl acceptor **2** by the general glycosylation method in 86% yield (β only) as a white amorphous solid. Analytical data for **24**: *R*_f_ 0.55 (ethyl acetate/hexanes, 1/1, v/v); [α]_D_^24^ −8.0 (*c* = 1.0, CHCl_3_); ^1^H NMR (400 MHz, CDCl_3_): δ 2.01 (s, 3H, CH_3_), 2.37 (m, 1H, COCH_2_C*H*a), 2.45–2.56 (m, 2H, COCH_2_C*H*b, COC*H*aCH_2_), 2.66 (m, 1H, COC*H*bCH_2_), 3.28 (s, 3H, OCH_3_), 3.33 (m, *J*_5′,6′_ = 9.7 Hz, 1H, H-5′), 3.39 (dd, *J*_4,5_ = 9.2 Hz, 1H, H-4), 3.46 (dd, *J*_2,3_ = 9.7 Hz, 1H, H-2), 3.51–3.73 (m, 5H, H-3′, 4′, 5, 6a, 6a′), 3.90 (dd, *J*_3,4_ = 8.8 Hz, 1H, H-3), 3.96 (m, 1H, H-6b), 4.05 (m, 1H, OC*H*aCH = CH_2_), 4.21–4.28 (m, 2H, H-6b′, OC*H*bCH = CH_2_), 4.43–4.48 (m, 2H, H-1′, C*H*Ph), 4.52 (d, *J*_1,2_ = 3.5 Hz, 1H, H-1), 4.58 (d, ^2^ *J* = 12.1 Hz, 1H, C*H*Ph), 4.72 (dd, ^2^ *J* = 11.2 Hz, 2H, C*H*_2_Ph), 4.78 (d, ^2^ *J* = 10.9 Hz, 1H, C*H*Ph), 4.90 (d, ^2^ *J* = 10.9 Hz, 1H, C*H*Ph), 4.96 (dd, *J*_2′,3′_ = 8.4 Hz, 1H, H-2′), 5.07 (m, 1H, CH=C*H*a), 5.15 (m, 1H, CH=C*H*b), 5.45 (s, 1H, >C*H*Ph), 5.71–5.86 (m, 1H, OCH_2_C*H*=CH_2_), 7.18–7.33 (m, 18H, aromatic), 7.37–7.43 (m, 2H, aromatic) ppm; ^13^C NMR (101 MHz, CDCl_3_): δ 27.9, 29.7 (×2), 29.8, 37.8, 55.2, 66.4, 68.2, 68.6, 69.6, 73.1, 73.3, 73.4, 74.9, 75.7, 77.2, 77.7, 78.7, 79.8, 81.2, 82.0, 98.1, 101.2, 101.4, 117.0, 126.0, 127.6, 127.7 (x 2), 127.9, 128.0 (×2), 128.2 (×2), 128.3 (×2), 128.4 (×2), 128.5 (×4), 129.0, 134.8, 137.2, 138.2, 138.3, 138.8, 171.1 ppm; HRMS (ESI/TOF) *m*/*z*: [M + Na]^+^ Calcd for C_49_H_56_NaO_13_ 875.3613; Found 875.3592.

#### Methyl 6-*O*-(2,3,4-Tri-*O*-benzoyl-α-l-rhamnopyranosyl)-2,3,4-tri-*O*-benzyl-α-d-glucopyranoside (**26**)

**26** was obtained from thioglycoside **25** and glycosyl acceptor **2** by the general glycosylation method in 98% yield (α only) as a white amorphous solid. Analytical data for **26** were in accordance with that reported previously.^117^

#### Methyl 2,3,4-Tri-*O*-benzyl-6-*O*-(2,3,4-tri-*O*-benzoyl-6-*O*-*p*-methoxybenzyl-β-d-glucopyranosyl)-α-d-glucopyranoside (**28**)

**28** was obtained from thioglycoside **27** and glycosyl acceptor **2** by the general glycosylation method in 68% yield (β only) as a white amorphous solid. Analytical data for **28** were in accordance with that reported previously.^105^

#### Methyl 2,3,4-Tri-*O*-benzyl-6-*O*-(3-*O*-benzyl-4,6-*O*-benzylidene-2-deoxy-2-phthalimido)-β-d-gIucopyranosyl-α-d-glucopyranoside (**32**)

**32** was obtained from thioglycoside **31** and glycosyl acceptor **2** by the general glycosylation method in 94% yield (β only) as a white amorphous solid. Analytical data for **32** were in accordance with that reported previously.^118^

#### Methyl 2,3,4-Tri-*O*-benzyl-6-*O*-(3,4,6-tri-*O*-benzoyl-2-deoxy-2-(2,2,2-trichloroethoxy)­carbamoyl-β-d-glucopyranosyl)-α-d-glucopyranoside (**34**)

**34** was obtained from thioglycoside **33** and glycosyl acceptor **2** by the general glycosylation method in 88% yield (β only) as a white amorphous solid. Analytical data for **34** were in accordance with that reported previously.^108^

#### Methyl *O*-(2,3,4,6-Tetra-*O-*benzoyl-β-d-galactopyranosyl)-(1→3)-*O*-(4,6-*O*-benzylidene-2-deoxy-2-phthalimido-β-d-glucopyranosyl)-(1→6)-2,3,4-tri-*O*-benzyl-α-d-glucopyranoside (**36**)

**36** was obtained from thioglycoside donor **35** and glycosyl acceptor **2** by the general glycosylation method in 70% yield (β only) as a white amorphous solid. Analytical data for **36**: *R*_f_ 0.30 (acetone/toluene, 1/9, v/v); [α]_D_^24^ + 42.7 (*c* = 1.0, CHCl_3_); ^1^H NMR (400 MHz, CDCl_3_): δ 3.12–3.19 (m, 4H, H-4, CH_3_), 3.35 (dd, 1H, *J*_2,3_ = 9.6 Hz, H-2), 3.55–3.61 (m, 2H, H-5, 6a), 3.65–3.71 (m, 1H, H-5′), 3.74–3.82 (m, 2H, H-3, 5″), 3.87–3.98 (m, 3H, H-4′, 6a′, C*H*Ph), 4.01–4.05 (dd, 1H, H-6b), 4.21–4.30 (m, 3H, 6a″, 6b″, C*H*Ph), 4.34–4.38 (m, 2H, H-1, 6b’), 4.45 (dd, *J*_2′,3′_ = 10.4 Hz, 1H, H-2′), 4.56 (d, 1H, ^2^ *J* = 12.1 Hz, C*H*Ph), 4.62 (d, 1H, ^2^ *J* = 10.9 Hz, C*H*Ph), 4.70 (d, 1H, C*H*Ph), 4.82–4.87 (m, 2H, H-3′, C*H*Ph), 4.93 (d, 1H, *J*_1″,2″_ = 8.1 Hz, H-1″), 5.21 (d, 1H, *J*_1′,2′_ = 8.5 Hz, H-1′), 5.33 (dd, 1H, *J*_3″,4″_ = 3.5 Hz, H-3″), 5.52 (dd, 1H, *J*_2″,3″_ = 10.3 Hz, H-2″), 5.60 (s, 1H, >C*H*Ph), 5.80 (dd, 1H, *J*_4″,5″_ = 1.2 Hz, H-4″), 6.90–8.01 (m, 44H, aromatic) ppm; ^13^C NMR (101 MHz, CDCl_3_): δ 21.5, 29.7, 54.9 (×2), 66.5, 67.6, 68.4, 68.7, 69.1, 70.2, 70.9, 71.9, 73.4, 74.6, 75.5, 76.2, 77.2, 77.5, 79.6, 81.2, 81.8, 97.9, 98.8, 100.7, 101.9, 125.3, 126.1 (×2), 127.5, 127.6 (×4), 127.8 (×3), 127.9, 128.1 (×4), 128.2 (×4), 128.3 (×3), 128.4 (×5), 128.5 (×2), 128.6 (×2), 128.7, 129.0 (×3), 129.3, 129.5 (×2), 129.6 (×2), 129.7 (×2), 130.0 (×2), 132.7, 133.1, 133.2, 133.4, 133.5, 136.9, 137.7, 138.1, 138.8, 164.6, 165.4, 165.5, 165.6 ppm; HRMS (ESI/TOF) *m*/*z*: [M + Na]^+^ Calcd for C_83_H_75_NO_21_Na 1444.4729; Found 1444.4746.

#### Methyl 2-*O*-(2,3,4,6-Tetra-*O*-benzyl-D-galactopyranosyl)-3,4,6-tri-*O*-benzyl-α-d-glucopyranoside (**38**)

**38** was obtained from thioglycoside **13** and glycosyl acceptor **37** by the general glycosylation method in 92% yield (α/β = 1/1.6) as a white amorphous solid. Analytical data for **38** were in accordance with that reported previously.^119^

#### Methyl 3-*O*-(2,3,4,6-Tetra-*O*-benzyl-d-galactopyranosyl)-2,4,6-tri-*O*-benzyl-α-d-glucopyranoside (**40**)

**40** was obtained from thioglycoside **13** and glycosyl acceptor **39** by general glycosylation method in 90% yield (α/β = 1/2.0) as a white amorphous solid. Analytical data for **40** were in accordance with that reported previously.^120^

#### Methyl 4-*O*-(2,3,4,6-Tetra-*O*-benzyl-d-galactopyranosyl)-2,3,6-tri-*O*-benzyl-α-d-glucopyranoside (**42**)

**42** was obtained from thioglycoside **13** and glycosyl acceptor **41** by general glycosylation method in 81% yield (α/β = 1/1.5) as a white amorphous solid. Analytical data for **42** were in accordance with that reported previously.^121^

#### Methyl 6-*O*-(2,3,4,6-Tetra-*O*-benzyl-d-galactopyranosyl)-2,3,4-tri-*O*-benzoyl-α-d-glucopyranoside (**44**)

**44** was obtained from thioglycoside **13** and glycosyl acceptor **43** by general glycosylation method in 89% yield (α/β = 1/1.6) as a white amorphous solid. Analytical data for **44** were in accordance with that reported previously.^122^

## Supplementary Material





## Data Availability

The data underlying this study are available in the published article and its Supporting Information.

## Notes

NMR spectra for all compounds (PDF)
Real-time video of the reaction (MP4)

## References

[1] Garegg P. J. (1997). Thioglycosides
as glycosyl donors in oligosaccharide synthesis. Adv. Carbohydr. Chem. Biochem..

[2] Codée J. D. C., Litjens R. E. J. N., van den Bos L. J., Overkleeft H. S., van der Marel G. A. (2005). Thioglycosides in sequential glycosylation
strategies. Chem. Soc. Rev..

[3] Zhong, W. ; Boons, G.-J. Glycoside Synthesis from 1-Sulfur/Selenium-Substituted Derivatives: Thioglycosides in Oligosaccharide Synthesis. In Handbook of Chemical Glycosylation; Demchenko, A. V. , Ed.; Wiley-VCH, 2008; pp 261–303.

[4] Lian G., Zhang X., Yu B. (2015). Thioglycosides
in Carbohydrate research. Carbohydr. Res..

[5] Fügedi P., Garegg P. J. (1986). A novel promoter
for the efficient construction of
1,2-trans linkages in glycoside synthesis, using thioglycosides as
glycosyl donors. Carbohydr. Res..

[6] Dasgupta F., Garegg P. J. (1988). Use of sulfenyl halides in carbohydrate
reactions.
Part I. Alkyl sulfenyl triflate as activator in the thioglycoside-mediated
formation of beta-glycosidic linkages during oligosaccharide synthesis. Carbohydr. Res..

[7] Crich D., Smith M. (2000). S-(4-Methoxyphenyl)
benzenethiosulfinate­(MPBT)/ trifluoromethanesulfonic
anhydride (Tf2O): a convenient system for the generation of glycosyl
triflates from thioglycosides. Org. Lett..

[8] Crich D., Smith M. (2001). 1-Benzenesulfinyl piperidine/trifluoromethanesulfonic
anhydride:
a potent combination of shelf-stable reagents for the low-temperature
conversion of thioglycosides to glycosyl triflates and for the formation
of diverse glycosidic linkages. J. Am. Chem.
Soc..

[9] Codée J. D. C., Litjens R. E. J. N., Heeten R., Overkleeft H. S., van Boom J. H., van der
Marel G. A. (2003). Ph_2_SO/Tf_2_O:
A powerful promotor system in chemoselective glycosylations using
thioglycosides. Org. Lett..

[10] Duron S. G., Polat T., Wong C. H. (2004). N-(Phenylthio)-e-caprolactam: A new
promoter for the activation of thioglycosides. Org. Lett..

[11] Veeneman G.
H., van Leeuwen S. H., van Boom J. H. (1990). Iodonium ion promoted reactions at
the anomeric centre. II. An efficient thioglycoside mediated approach
toward the formation of 1,2-trans linked glycosides and glycosidic
esters. Tetrahedron Lett..

[12] Nicolaou K. C., Seitz S. P., Papahatjis D. P. (1983). A mild
and general method for the
synthesis of O-glycosides. J. Am. Chem. Soc..

[13] Kihlberg J. O., Leigh D. A., Bundle D. R. (1990). The in situ activation of thioglycosides
with bromine: an improved glycosylation method. J. Org. Chem..

[14] Ravindranathan
Kartha K., Aloui M., Field R. A. (1996). Iodine: a versatile
reagent in carbohydrate chemistry II. Efficient chemospecific activation
of thiomethylglycosides. Tetrahedron Lett..

[15] Burkart M. D., Zhang Z., Hung S.-C., Wong C.-H. (1997). A new method for
the synthesis of fluoro-carbohydrates and glycosides using selectfluor. J. Am. Chem. Soc..

[16] Ercegovic T., Meijer A., Magnusson G., Ellervik U. (2001). Iodine monochloride/silver
trifluoromethanesulfonate (ICI/AgOTf) as a convenient promoter system
for O-glycoside synthesis. Org. Lett..

[17] Wever W. J., Cinelli M. A., Bowers A. A. (2013). Visible
Light Mediated Activation
and O-Glycosylation of Thioglycosides. Org.
Lett..

[18] Mao R.-Z., Guo F., Xiong D.-C., Li Q., Duan J., Ye X.-S. (2015). Photoinduced
C-S Bond Cleavage of Thioglycosides and Glycosylation. Org. Lett..

[19] Spell M. L., Deveaux K., Bresnahan C. G., Bernard B. L., Sheffield W., Kumar R., Ragains J. R. (2016). A Visible-Light-Promoted
O-Glycosylation
with a Thioglycoside Donor. Angew. Chem., Int.
Ed..

[20] Mao R.-Z., Xiong D.-C., Guo F., Li Q., Duan J., Ye X.-S. (2016). Light-driven highly efficient glycosylation reactions. Org. Chem. Front..

[21] Escopy S., Demchenko A. V. (2022). Transition-Metal-Mediated
Glycosylation with Thioglycosides. Chem. –
Eur. J..

[22] Ferrier R. J., Hay R. W., Vethaviyasar N. (1973). A potentially
versatile synthesis
of glycosides. Carbohydr. Res..

[23] Van
Cleve J. W. (1979). Reinvestigation of the preparation of cholesteryl 2,
3, 4, 6-tetra-O-benzyl-α-D-glucopyranoside. Carbohydr. Res..

[24] Hanessian S., Bacquet C., Lehong N. (1980). Chemistry of the glycosidic
linkage.
Exceptionally fast and efficient formation of glycosides by remote
activation. Carbohydr. Res..

[25] Garegg P. J., Henrichson C., Norberg T. (1983). A reinvestigation of
glycosidation
reactions using 1-thioglycosides as glycosyl donors and thiophilic
cations as promoters. Carbohydr. Res..

[26] Mukaiyama T., Nakatsuka T., Shoda S. I. (1979). An efficient glucosylation of alcohol
using 1-thioglucoside derivative. Chem. Lett..

[27] Demchenko A. V., Malysheva N. N., De Meo C. (2003). S-Benzoxazolyl (SBox) glycosides
as novel, versatile glycosyl donors for stereoselective 1,2-cis glycosylation. Org. Lett..

[28] Demchenko A. V., Kamat M. N., De Meo C. (2003). S-Benzoxazolyl (SBox) glycosides
in oligosaccharide synthesis: novel glycosylation approach to the
synthesis of b-D-glucosides, b-D-galactosides, and a-D-mannosides. Synlett.

[29] Woodward R. B., Logusch E., Nambiar K. P., Sakan K., Ward D. E., Au-Yeung B. W., Balaram P., Browne L. J., Card P. J., Chen C. H. (1981). Asymmetric total synthesis of erythromycin. 3. Total
synthesis of erythromycin. J. Am. Chem. Soc..

[30] Hanessian S., Ugolini A., Dube D., Hodges P. J., Andre C. (1986). Synthesis
of (+)-avermectin B1a. J. Am. Chem. Soc..

[31] Lear M. J., Yoshimura F., Hirama M. (2001). A direct and efficient a-selective
glycosylation protocol for the kedarcidin sugar, L-mycarose: AgPF_6_ as a remarkable activator of 2-deoxythioglycosides. Angew. Chem., Int. Ed..

[32] Kamat M. N., Rath N. P., Demchenko A. V. (2007). Versatile
synthesis and mechanism
of activation of S-benzoxazolyl glycosides. J. Org. Chem..

[33] Hasty S. J., Ranade S. C., Demchenko A. V. (2014). A study of silver­(I) perchlorate
as an effective promoter for chemical glycosylation with thioimidates
and thioglycosides. Rep. Org. Chem..

[34] Kaeothip S., Pornsuriyasak P., Rath N. P., Demchenko A. V. (2009). Unexpected
orthogonality of S-benzoxazolyl and S-thiazolinyl derivatives: application
to expeditious oligosaccharide assembly. Org.
Lett..

[35] Dondoni A., Marra A., Massi A. (2005). Hybrid solution/solid-phase
synthesis
of oligosaccharides by using trichloroacetyl isocyanate as sequestration-enabling
reagent of sugar alcohols. Angew. Chem., Int.
Ed..

[36] Pooladian F., Escopy S., Demchenko A. V. (2022). Activation of thioglycosides with
copper­(II) bromide. Molecules.

[37] Goswami M., Ellern A., Pohl N. L. (2013). Bismuth­(V)-mediated
thioglycoside
activation. Angew. Chem., Int. Ed..

[38] Goswami M., Ashley D. C., Baik M. H., Pohl N. L. (2016). Mechanistic Studies
of Bismuth­(V)-Mediated Thioglycoside Activation Reveal Differential
Reactivity of Anomers. J. Org. Chem..

[39] Kabotso D. E. K., Pohl N. L. B. (2017). Pentavalent bismuth as a universal promoter for S-containing
glycosyl donors with a thiol additive. Org.
Lett..

[40] Kumar V., Yadav N., Kartha K. P. (2014). In­(III) triflate-catalyzed
detritylation
and glycosylation by solvent-free ball milling. Carbohydr. Res..

[41] Vibhute A. M., Dhaka A., Athiyarath V., Sureshan K. M. (2016). A versatile glycosylation
strategy via Au (III) catalyzed activation of thioglycoside donors. Chem. Sci..

[42] Zhu X., Schmidt R. R. (2009). New principles for
glycoside-bond formation. Angew. Chem., Int.
Ed..

[43] Escopy S., Singh Y., Demchenko A. V. (2021). Palladium­(II)-assisted activation
of thioglycosides. Org. Biomol. Chem..

[44] Ridgway L. M., Das A., Shadrick M. L., Demchenko A. V. (2024). Ferric Chloride Promoted Glycosidation
of Alkyl Thioglycosides. Molecules.

[45] Paulsen H. (1982). Advances in
selective chemical syntheses of complex oligosaccharides. Angew. Chem., Int. Ed..

[46] Mootoo D. R., Konradsson P., Udodong U., Fraser-Reid B. (1988). ″Armed″
and ″disarmed″ n-pentenyl glycosides in saccharide couplings
leading to oligosaccharides. J. Am. Chem. Soc..

[47] Fraser-Reid B., Udodong U. E., Wu Z. F., Ottosson H., Merritt J. R., Rao C. S., Roberts C., Madsen R. (1992). n-Pentenyl glycosides
in organic chemistry: a contemporary example of serendipity. Synlett.

[48] Fraser-Reid B., Wu Z., Andrews C. W., Skowronski E., Bowen J. P. (1991). Torsional effects
in glycoside reactivity: saccharide couplings mediated by acetal protecting
groups. J. Am. Chem. Soc..

[49] Wilson B. G., Fraser-Reid B. (1995). n-Pentenyl glycoside based methodology for determining
the relative reactivities of variously protected pairs of glycosides. J. Org. Chem..

[50] Douglas N. L., Ley S. V., Lucking U., Warriner S. L. (1998). Tuning glycoside
reactivity: new tool for efficient oligosaccharides synthesis. J. Chem. Soc., Perkin Trans. 1.

[51] Zhang Z., Ollmann I. R., Ye X. S., Wischnat R., Baasov T., Wong C. H. (1999). Programmable one-pot oligosaccharide synthesis. J. Am. Chem. Soc..

[52] Fridman M., Solomon D., Yogev S., Baasov T. (2002). One-pot synthesis of
glucosamine oligosaccharides. Org. Lett..

[53] Bandara, M. D. ; Yasomanee, J. P. ; Demchenko, A. V. Application of Armed, Disarmed, Superarmed and Superdisarmed Building Blocks in Stereocontrolled Glycosylation and Expeditious Oligosaccharide Synthesis. In Selective Glycosylations: Synthetic Methods and Catalysts; Bennett, C. S. , Ed.; Wiley, 2017; pp 29–58.

[54] Kamat M. N., Demchenko A. V. (2005). Revisiting
the armed-disarmed concept rationale: chemoselective
activation of the S-benzoxazolyl glycosides in oligosaccharide synthesis. Org. Lett..

[55] Mydock L. K., Demchenko A. V. (2008). Superarming the S-benzoxazolyl glycosyl donors by simple
2-O-benzoyl-3,4,6-tri-O-benzyl protection. Org.
Lett..

[56] Mydock L.
K., Demchenko A. V. (2008). Application
of the superarmed glycosyl donor to chemoselective
oligosaccharide synthesis. Org. Lett..

[57] Premathilake H. D., Mydock L. K., Demchenko A. V. (2010). Superarming
common glycosyl donors
by simple 2-O-benzoyl-3,4,6-tri-O-benzyl protection. J. Org. Chem..

[58] Zhu T., Boons G. J. (2001). Thioglycosides protected as trans-2,3-cyclic carbonates
in chemoselective glycosylations. Org. Lett..

[59] Jensen H.
H., Pedersen C. M., Bols M. (2007). Going to extremes: “super”
armed glycosyl donors in glycosylation chemistry. Chem. – Eur. J..

[60] Pedersen C. M., Nordstrom L. U., Bols M. (2007). “Super armed” glycosyl
donors: conformational arming of thioglycosides by silylation. J. Am. Chem. Soc..

[61] Heuckendorff M., Premathilake H. D., Pornsuriyasak P., Madsen A. Ø., Pedersen C. M., Bols M., Demchenko A. V. (2013). Superarming
of glycosyl donors by combined neighboring and conformational effects. Org. Lett..

[62] Bandara M. D., Yasomanee J. P., Rath N. P., Pedersen C. M., Bols M., Demchenko A. V. (2017). Conformationally superarmed S-ethyl glycosyl donors
as effective building blocks for chemoselective oligosaccharide synthesis
in one pot. Org. Biomol. Chem..

[63] Panza M., Civera M., Yasomanee J. P., Belvisi L., Demchenko A. V. (2019). Bromine-promoted
glycosidation of conformationally superarmed thioglycosides. Chem. – Eur. J..

[64] Geringer S. A., Demchenko A. V. (2018). Iron­(III) chloride-catalyzed activation of glycosyl
chlorides. Org. Biomol. Chem..

[65] Morelli L., Bernardi A., Sattin S. (2014). Synthesis
of potential allosteric
modulators of Hsp90 by chemical glycosylation of Eupomatenoid-6. Carbohydr. Res..

[66] Gao Y., Guery J., Jacoboni C. (1993). FeCl3 behavior in acetonitrile: structures
of [FeCl2­(CH3CN)­4]­[FeCl4] and [AlCl­(CH3CN)­5]­[FeCl4]­2.CH3CN. Acta Crystallogr., Sect. C.

[67] Cotton S. A., Franckevicius V., Fawcett J. (2002). Syntheses and structures of iron­(III)
complexes of simple N-donor ligands. Polyhedron.

[68] Singh Y., Demchenko A. V. (2020). Defining
the scope of the acid-catalyzed glycosidation
of glycosyl bromides. Chem. – Eur. J..

[69] Sawama Y., Masuda M., Asai S., Goto R., Nagata S., Nishimura S., Monguchi Y., Sajiki H. (2015). FeCl3-catalyzed
self-cleaving
deprotection of methoxyphenylmethyl-protected alcohols. Org. Lett..

[70] Geringer S. A., Mannino M. P., Bandara M. D., Demchenko A. V. (2020). Picoloyl
protecting group in synthesis: focus on a highly chemoselective catalytic
removal. Org. Biomol. Chem..

[71] Bauer I., Knolker H. J. (2015). Iron catalysis in
organic synthesis. Chem. Rev..

[72] Huang T. Y., Zulueta M. M., Hung S. C. (2014). Regioselective
one-pot protection,
protection-glycosylation and protection-glycosylation-glycosylation
of carbohydrates: a case study with D-glucose. Org. Biomol. Chem..

[73] Gouasmat A., Lemétais A., Solles J., Bourdreux Y., Beau J.-M. (2017). Catalytic Iron­(III) Chloride Mediated Site-Selective
Protection of Mono- and Disaccharides and One Trisaccharide. Eur. J. Org. Chem..

[74] Kiso M., Anderson L. (1979). The ferric chloride-catalyzed
glycosylation of alcohols
by 2-acylamido-2-deoxy-β-D-glucopyranose 1-acetates. Carbohydr. Res..

[75] Kiso M., Anderson L. (1985). The synthesis of disaccharides by
the ferric chloride-catalyzed
coupling of 2-acylamido-2-deoxy-b-D-glucopyranose 1-acetates to protected
sugar acceptors. Carbohydr. Res..

[76] Kiso M., Nishiguchi H., Hasegawa A. (1980). Application of ferric chloride-catalyzed
glycosylation to a synthesis of glycolipids. Carbohydr. Res..

[77] Dasgupta F., Garegg P. J. (1989). Synthesis of ethyl and
phenyl 1-thio-1,2-trans-D-glycopyranosides
from the corresponding per-O-acetylated glycopyranoses having a 1,2-trans-configuration
using anhydrous ferric chloride as a promoter. Acta Chem. Scand..

[78] Lerner L. M. (1990). Ferric
chloride-molecular sieve-catalyzed formation of a nonreducing disaccharide
derivative. Carbohydr. Res..

[79] Chatterjee S. K., Nuhn P. (1998). Stereoselective a-glycosidation
using FeCl_3_ as a Lewis
acid catalyst. Chem. Commun..

[80] Seibel J., Hillringhaus L., Moraru R. (2005). Microwave-assisted glycosylation
for the synthesis of glycopeptides. Carbohydr.
Res..

[81] Wei G., Lv X., Du Y. (2008). FeCl3-catalyzed alpha-glycosidation
of glycosamine
pentaacetates. Carbohydr. Res..

[82] Narayanaperumal S., César da Silva R., Monteiro J. L., Corrêa A. G., Paixão M. W. (2012). Iron­(III) Chloride Catalyzed Glycosylation of Peracylated
Sugars with Allyl/Alkynyl Alcohols. J. Braz.
Chem. Soc..

[83] Marzag H., Robert G., Dufies M., Bougrin K., Auberger P., Benhida R. (2015). FeCl3-promoted and
ultrasound-assisted synthesis of
resveratrol O-derived glycoside analogs. Ultrason.
Sonochem..

[84] Laursen J. B., Petersen L., Jensen K. J. (2001). Intramolecular glycosylation under
neutral conditions for synthesis of 1,4-linked disaccharides. Org. Lett..

[85] Rasmussen M. R., Marqvorsen M. H., Kristensen S. K., Jensen H. H. (2014). A protocol for metal
triflate catalyzed direct glycosylations with GalNAc 1-OPiv donors. J. Org. Chem..

[86] Shetye G. S., Singh N., Jia C., Nguyen C. D., Wang G., Luk Y. Y. (2014). Specific maltose
derivatives modulate the swarming
motility of nonswarming mutant and inhibit bacterial adhesion and
biofilm formation by Pseudomonas aeruginosa. ChemBioChem.

[87] Mukherjee M. M., Basu N., Ghosh R. (2016). Iron­(III) chloride modulated selective
1,2-trans glycosylation based on glycosyl trichloroacetimidate donors
and its application in orthogonal glycosylation. RSC Adv..

[88] Yang F., Hou W., Zhu D., Tang Y., Yu B. (2022). A Stereoselective Glycosylation
Approach to the Construction of 1,2-trans-beta-d-Glycosidic Linkages
and Convergent Synthesis of Saponins. Chem.
– Eur. J..

[89] Sun G., Wu Y., Liu A., Qiu S., Zhang W., Wang Z., Zhang J. (2018). Substoichiometric FeCl3 Activation of Propargyl Glycosides for the
Synthesis of Disaccharides and Glycoconjugates. Synlett.

[90] Sail D., Kovac P. (2012). Benzoylated ethyl 1-thioglycosides: direct preparation from per-O-benzoylated
sugars. Carbohydr. Res..

[91] Ranade S. C., Kaeothip S., Demchenko A. V. (2010). Glycosyl
alkoxythioimidates as complementary
building blocks for chemical glycosylation. Org. Lett..

[92] Chatterjee S., Moon S., Hentschel F., Gilmore K., Seeberger P. H. (2018). An Empirical
Understanding of the Glycosylation Reaction. J. Am. Chem. Soc..

[93] Grube M., Lee B.-Y., Garg M., Michel D., Vilotijević I., Malik A., Seeberger P. H., Varón Silva D. (2018). Synthesis
of Galactosylated Glycosylphosphatidylinositol Derivatives from Trypanosoma
brucei. Chem. – Eur. J..

[94] Forsythe N. P., Mize E. R., Kashiwagi G. A., Demchenko A. V. (2024). Expedient
synthesis of superarmed glycosyl donors via oxidative thioglycosidation
of glycals. Synthesis.

[95] Andersson F., Fugedi P., Garegg P. J., Nashed M. (1986). Synthesis of 1,2-cis-linked
glycosides using dimethyl­(methylthio)­sulfonium triflate as promoter
and thioglycosides as glycosyl donors. Tetrahedron
Lett..

[96] Ekelöf K., Oscarson S. (1996). Synthesis of oligosaccharide structures
from the lipopolysaccharide
of *Moraxella catarrhalis*. J.
Org. Chem..

[97] Fekete A., Borbas A., Antus S., Liptak A. (2009). Synthesis of 3,6-branched
arabinogalactan-type tetra- and hexasaccharides for characterization
of monoclonal antibodies. Carbohydr. Res..

[98] Käsbeck L., Kessler H. (1997). Convenient Syntheses of 2,3,4,6-Tetra-O-alkylated D-Glucose
and D-Galactose. Liebigs Ann./Recueil.

[99] Mydock L. K., Kamat M. N., Demchenko A. V. (2011). Direct
synthesis of diastereomerically
pure glycosyl sulfonium salts. Org. Lett..

[100] Elie C., Verduyn R., Dreef C., Brounts D., van der Marel G., van Boom J. (1990). Synthesis of 6–0-(α-D-mannopyranosyl)-D-myo-inositol:
a fragment from mycobacteria phospholipids. Tetrahedron.

[101] Sianturi J., Priegue P., Hu J., Yin J., Seeberger P. H. (2022). Semi-Synthetic Glycoconjugate Vaccine Lead Against
Acinetobacter baumannii 17978. Angew. Chem.,
Int. Ed..

[102] Garegg P. J., Kvarnstrom I., Niklasson A., Niklasson G., Svensson S. C. T. (1993). Partial substitution of thioglycosides
by phase transfer catalyzed benzoylation and benzylation. J. Carbohydr. Chem..

[103] Parsons T. B., Moir J. W. B., Fairbanks A. J. (2009). Synthesis
of a truncated bi-antennary complex-type N-glycan oxazoline; glycosylation
catalysed by the endohexosaminidases Endo A and Endo M. Org. Biomol. Chem..

[104] Krumb M., Lucas T., Opatz T. (2019). Visible Light
Enables
Aerobic Iodine Catalyzed Glycosylation. Eur.
J. Org. Chem..

[105] Fujikawa K., Vijaya Ganesh N., Tan Y. H., Stine K. J., Demchenko A. V. (2011). Reverse
orthogonal approach to oligosaccharide synthesis. Chem. Commun..

[106] Smoot J. T., Demchenko A. V. (2008). How the
arming participating moieties
can broaden the scope of chemoselective oligosaccharide synthesis
by allowing the inverse armed-disarmed approach. J. Org. Chem..

[107] Kerékgyártó J., van der Ven J. G. M., Kamerling J. P., Lipták A., Vliegenthart J. F. G. (1993). Synthesis
of a selectively protected trisaccharide building block that is part
of xylose-containing carbohydrate chains from N-glycoproteins. Carbohydr. Res..

[108] Kamkhachorn T., Parameswar A. R., Demchenko A. V. (2010). Comparison
of the armed/disarmed building blocks of the D-gluco and D-glucosamino
series in the context of chemoselective oligosaccharide synthesis. Org. Lett..

[109] Pooladian F., Das A., Demchenko A. V. (2025). Chemical
Synthesis of Two Fucosylated Human Milk Oligosaccharides: 3-Fucosyllactose
and Lacto-N-fucopentaose V. Chem. – Eur.
J..

[110] Shrestha G., Kashiwagi G. A., Stine K. J., Demchenko A. V. (2022). Streamlined
access to carbohydrate building blocks: Methyl 2,4,6-tri-O-benzyl-alpha-d-glucopyranoside. Carbohydr. Res..

[111] Zhang F., Zhang W., Zhang Y., Curran D. P., Liu G. (2009). Synthesis and Applications of a Light-Fluorous Glycosyl Donor. J. Org. Chem..

[112] Codée J. D., Van den Bos L. J., Litjens R. E. J. N., Overkleeft H. S., Van Boeckel C. A. A., Van Boom J. H., Van der
Marel G. A. (2004). Chemoselective glycosylations using sulfonium triflate
activator systems. Tetrahedron.

[113] Nguyen H. M., Chen Y. N., Duron S. G., Gin D. Y. (2001). Sulfide-mediated
dehydrative glycosylation. J. Am. Chem. Soc..

[114] Mathew F., Jayaprakash K., Fraser-Reid B., Mathew J., Scicinski J. (2003). Microwave-assisted
saccharide coupling
with n-pentenyl glycosyl donors. Tetrahedron
Lett..

[115] Lu S. R., Lai Y. H., Chen J. H., Liu C. Y., Mong K. K. (2011). Dimethylformamide: an unusual glycosylation modulator. Angew. Chem., Int. Ed..

[116] He H., Zhu X. (2014). Thioperoxide-mediated
activation of thioglycoside donors. Org. Lett..

[117] Hu Y., Yu K., Shi L. L., Liu L., Sui J. J., Liu D. Y., Xiong B., Sun J. S. (2017). o-(p-Methoxyphenylethynyl)­phenyl
Glycosides: Versatile New Glycosylation Donors for the Highly Efficient
Construction of Glycosidic Linkages. J. Am.
Chem. Soc..

[118] Bai Y., Boons G.-J., Burton A., Johnson M., Haller M. (2000). Vinyl Glycosides
in Oligosaccharide Synthesis (Part 6): 3-Buten-2-YL 2-Azido-2-Deoxy
Glycosides and 3-Buten-2-YL 2-Phthalimido-2-Deoxy Glycosides as Novel
Glycosyl Donors. J. Carbohydr. Chem..

[119] Premathilake H. D., Demchenko A. V. (2012). 2-Allylphenyl
glycosides as complementary
building blocks for oligosaccharide and glycoconjugate synthesis. Beilstein J. Org. Chem..

[120] Kobashi Y., Mukaiyama T. (2005). Glycosyl phosphonium halide as a
reactive intermediate in highly a-selective glycosylation. Bull. Chem. Soc. Jpn..

[121] Wegmann B., Schmidt R. R. (1987). Glycosylimidates. 27. The application
of the trichloroacetimidate method to the synthesis of a-D-glucopyranosides
and a-D-galactopyranosides. J. Carbohydr. Chem..

[122] Dent A., Escopy S., Demchenko A. V. (2023). Cooperatively
Catalyzed Activation of Thioglycosides That Bypasses Intermediacy
of Glycosyl Halides. Chem. – Eur. J..

