# Supplementary material for: Activation of Thioglycosides with Ferric Chloride: Scope and Mechanism
Source: ACS Omega. 2026 May 21;11(22):33123–33. doi: 10.1021/acsomega.6c03149 (PMC13261420; doi:10.1021/acsomega.6c03149)
Supplement: Supplementary file 1 [file ao6c03149_si_001.pdf]

Supporting Information

Activation of Thioglycosides with Ferric Chloride: Scope and Mechanism

Lacie M. Ridgway, Rachel L. Turecki, Faranak Pooladian, Anupama Das, and Alexei V. Demchenko*

Department of Chemistry, Saint Louis University, 3501 Laclede Ave, St. Louis, Missouri 63103, USA; e-mail: alexei.demchenko@slu.edu

Contents:

**NMR Spectra of new compounds 24 and 36**	**S2**
**NMR Spectra of known compounds**	**S8**

NMR Spectra of new compounds of 24 and 36

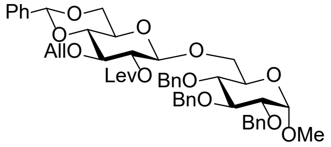

24

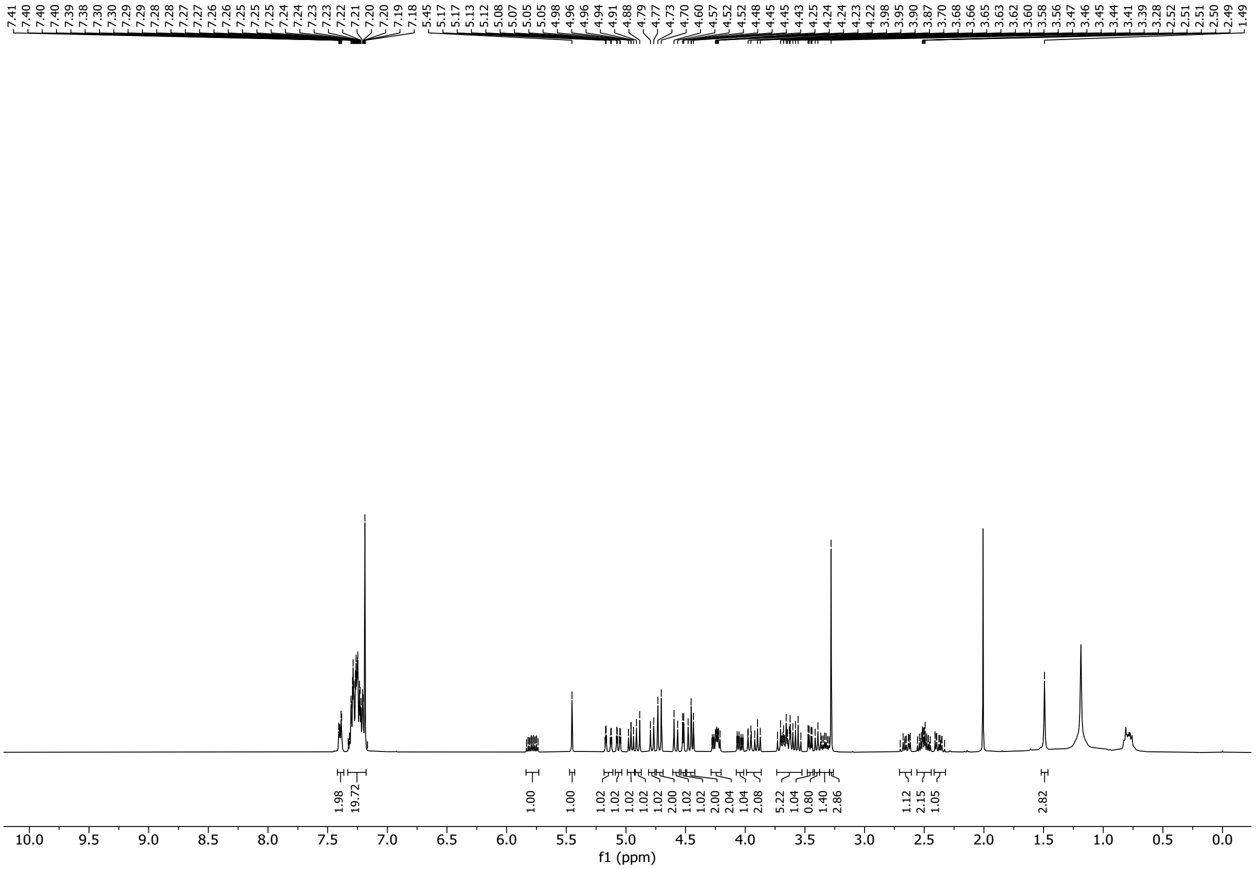

Figure S1. ^{1}H NMR spectrum (400 MHz, $CDCl_3$) of compound 24

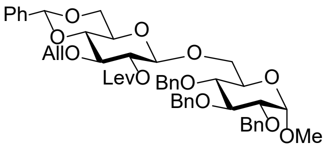

24

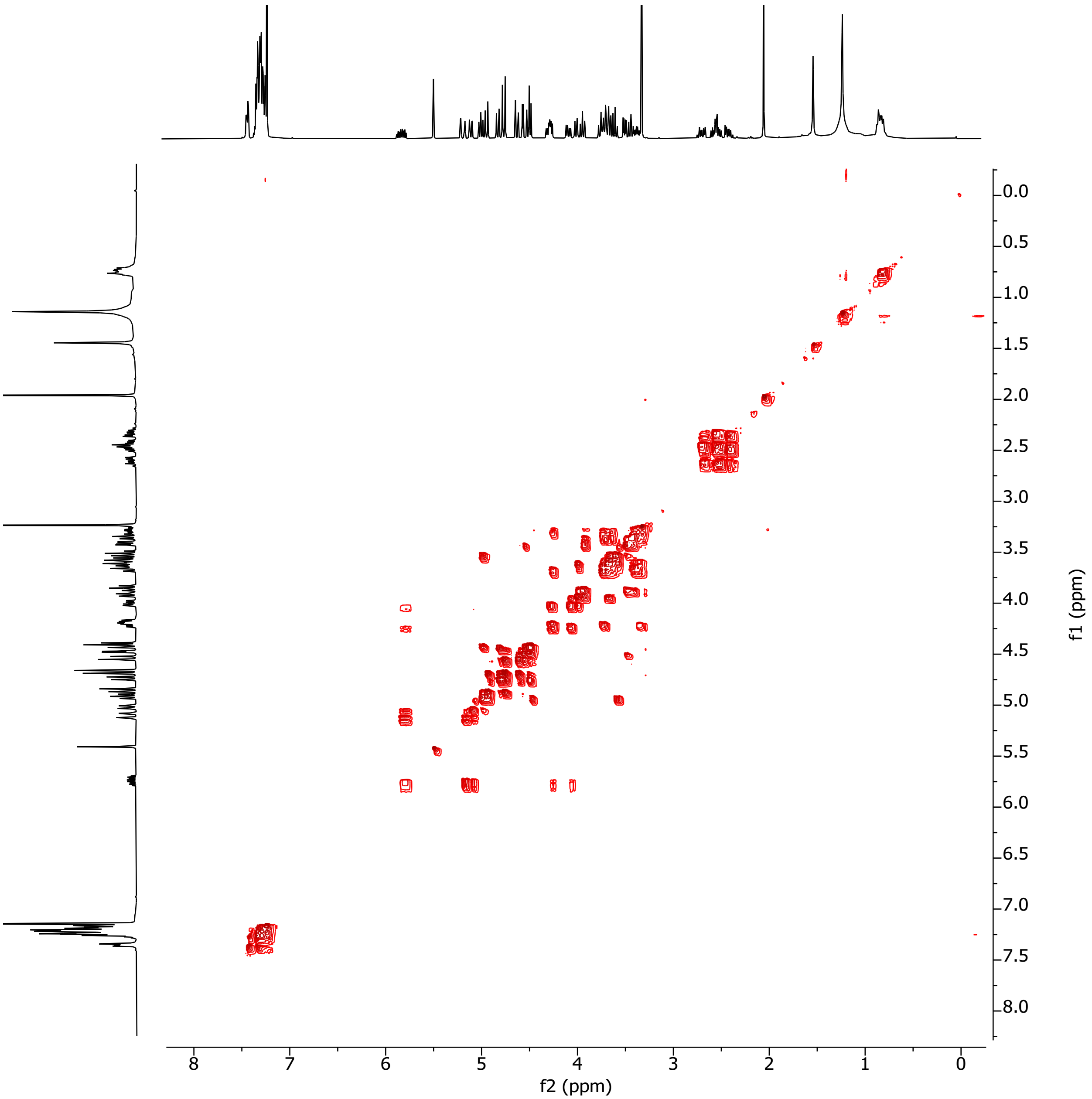

Figure S2. COSY NMR spectrum (400 MHz, CDCl₃) of compound 24

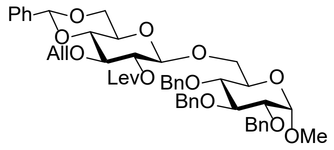

24

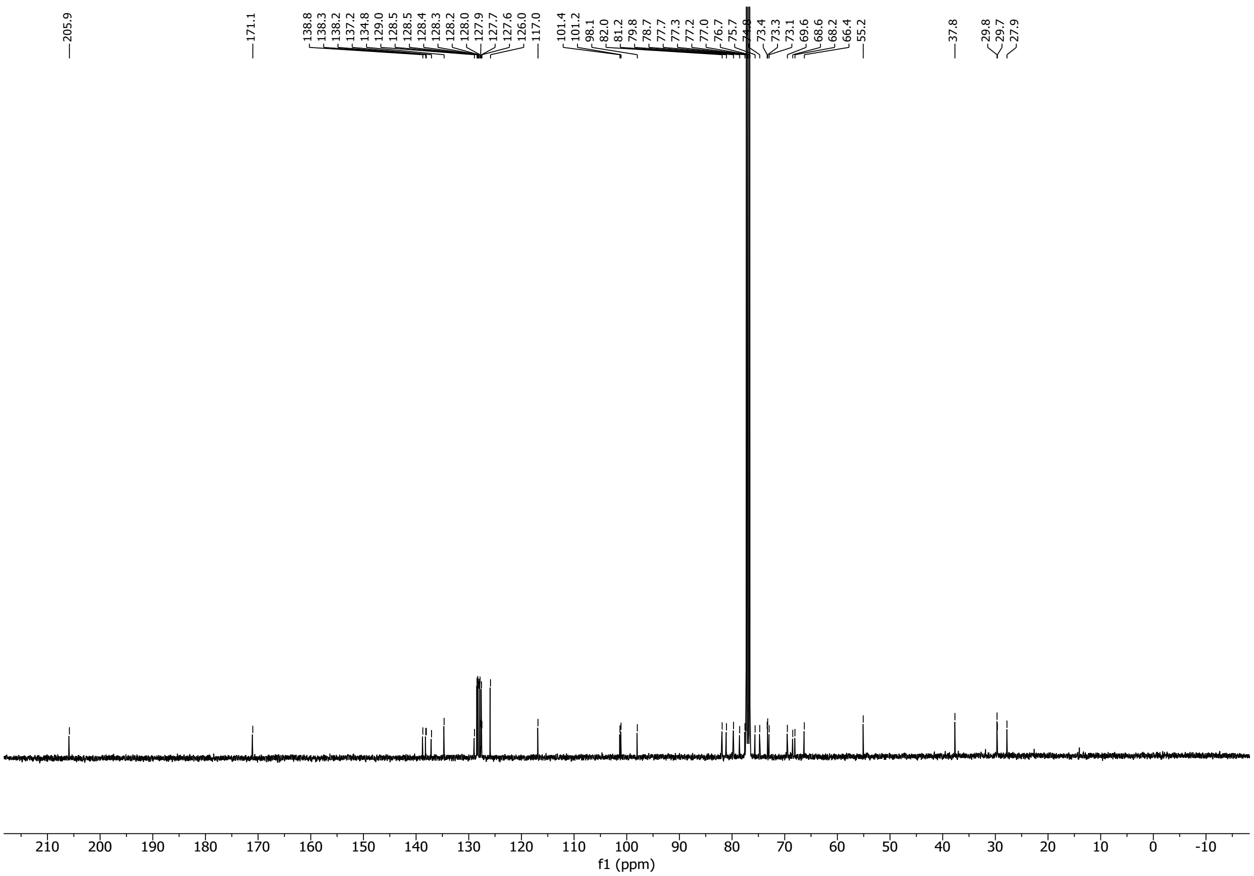

Figure S3. ^{13}C NMR spectrum (101 MHz, CDCl3) of compound 24

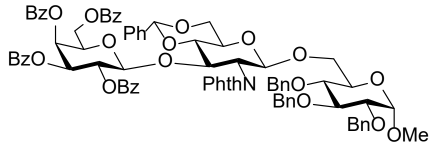

36

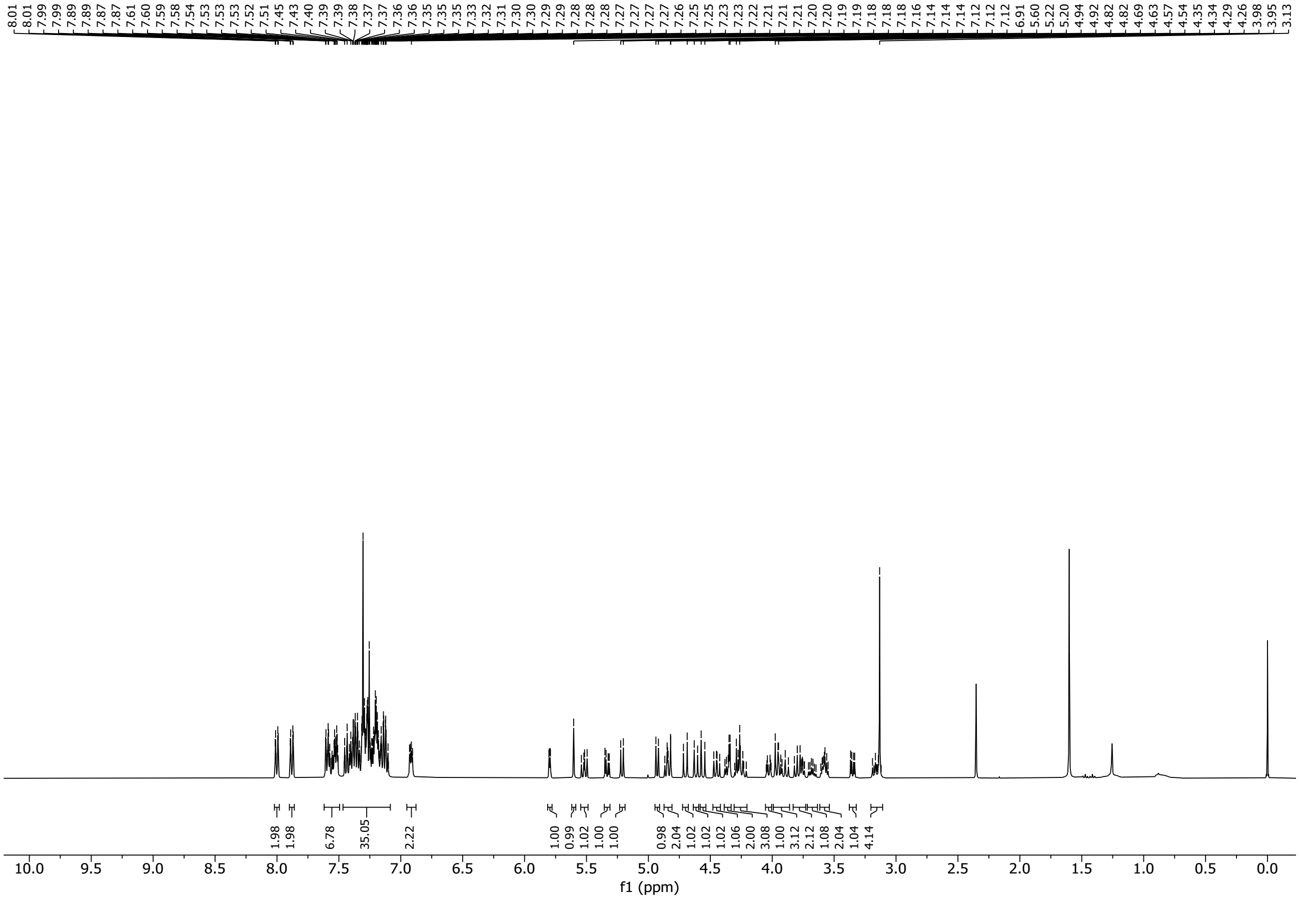

Figure S4. [1]**H NMR spectrum (400 MHz, $CDCl_3$) of compound 36**

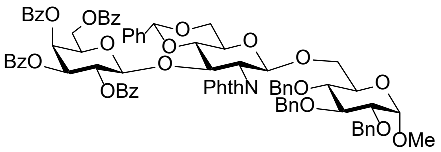

36

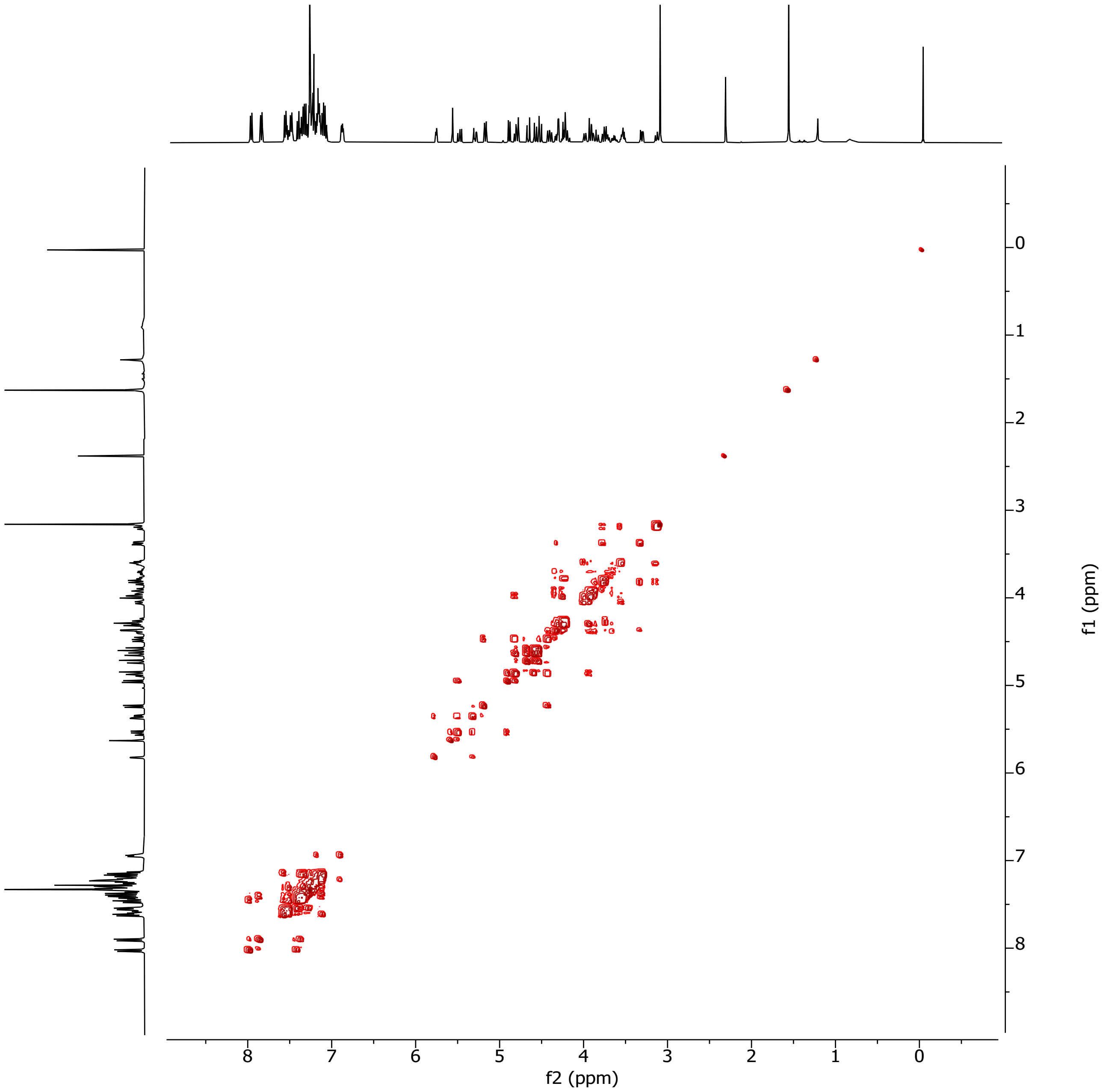

Figure S5. COSY 2D NMR spectrum (400 MHz, $CDCl_3$) of compound 36

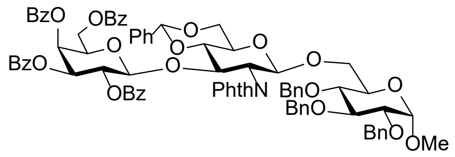

36

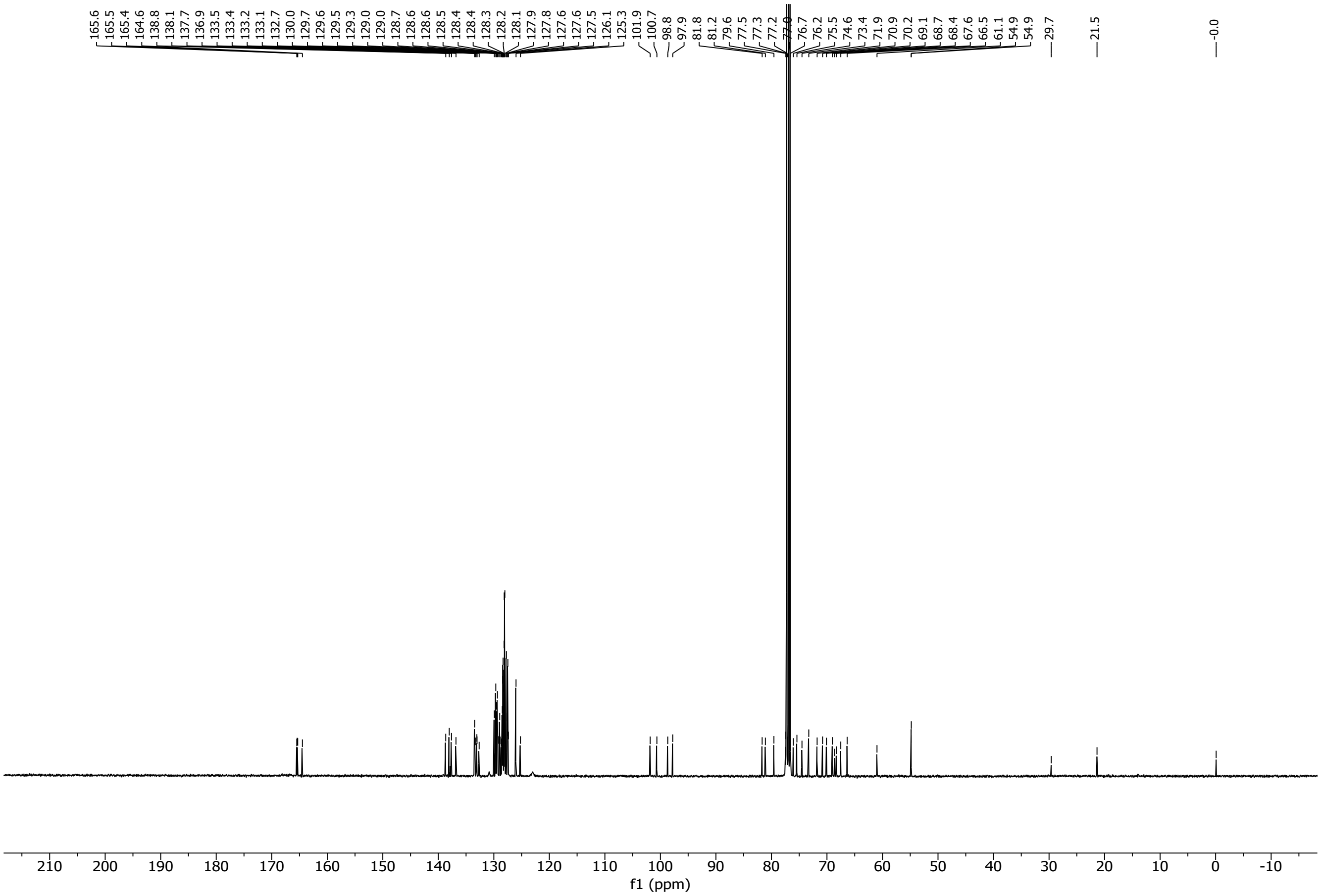

Figure S6. ^{13}**C NMR spectrum (101 MHz, CDCl**$_3$**) of compound 36**

NMR Spectra of known compounds

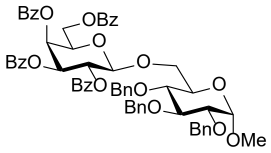

3

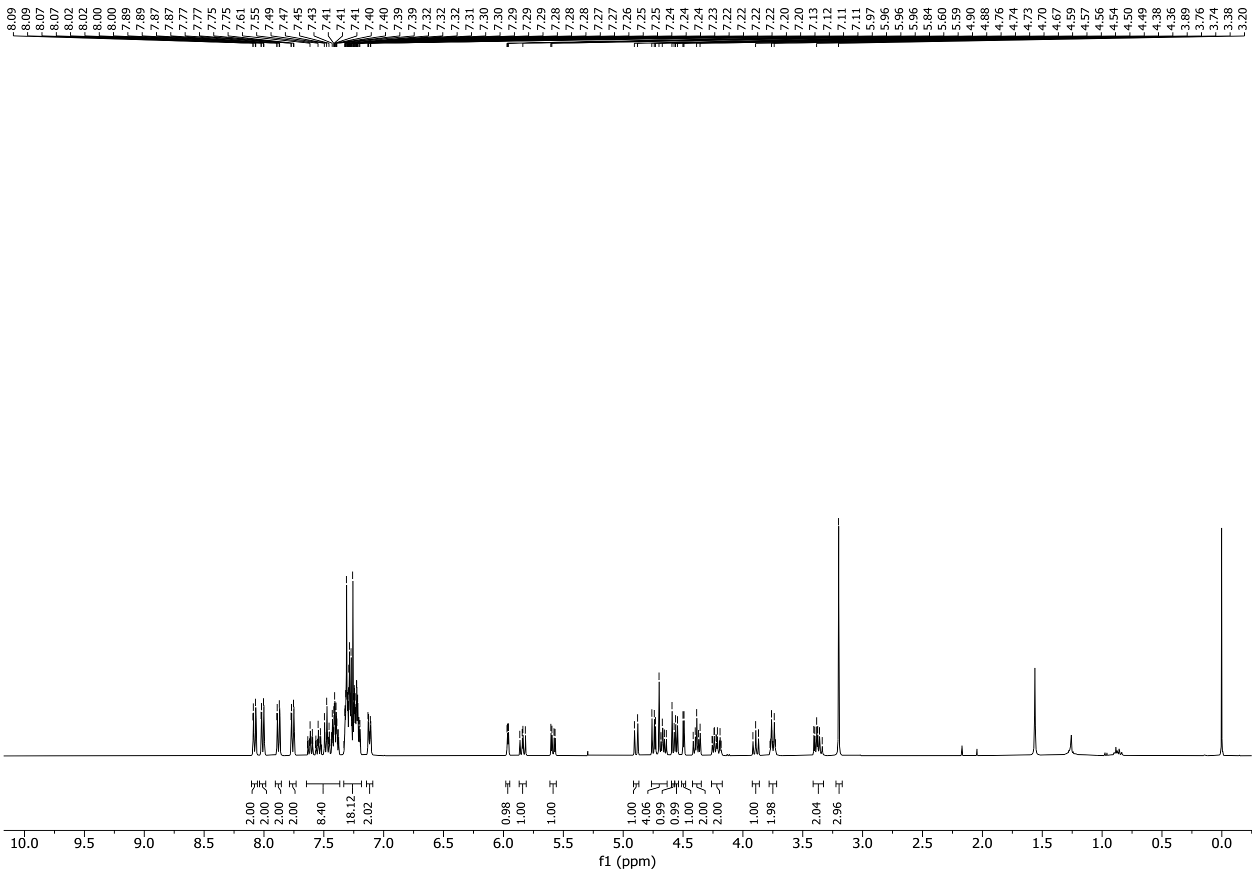

Figure S7. **^{1}H NMR spectrum (400 MHz, $CDCl_3$) of compound 3**

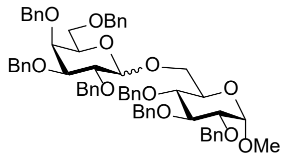

5

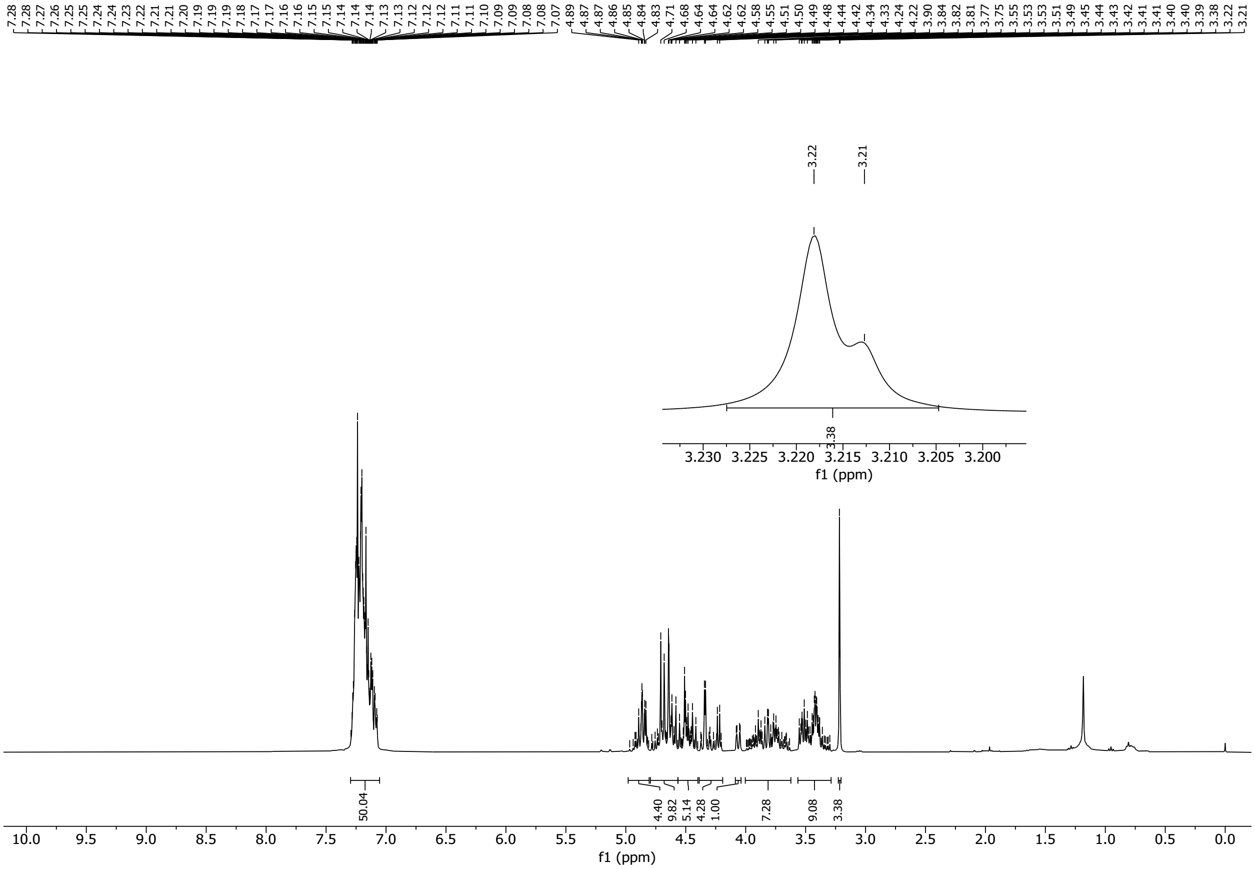

Figure S8. ^{1}H NMR spectrum (400 MHz, $CDCl_3$) of compound 5

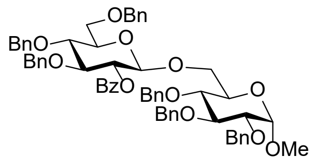

11

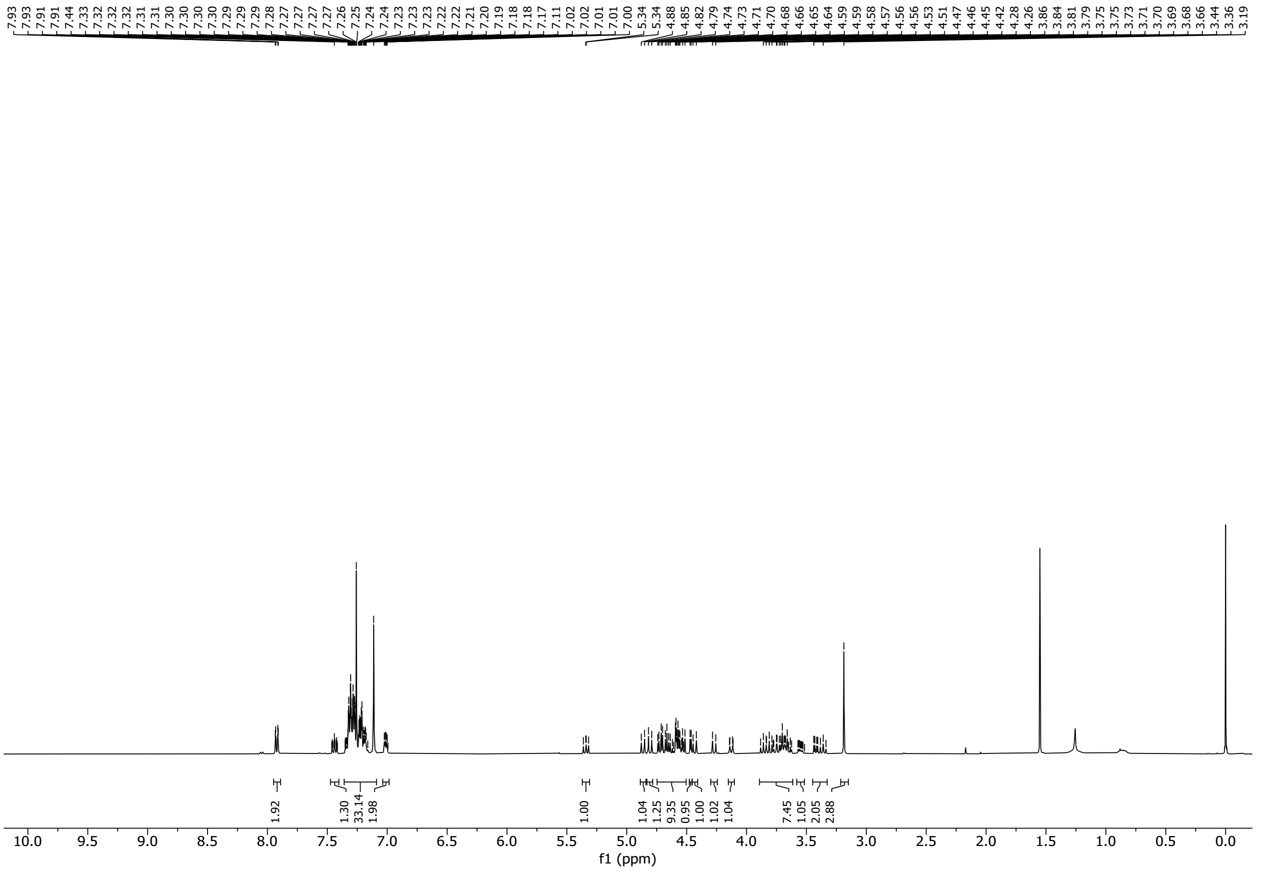

Figure S9. ^{1}H NMR spectrum (400 MHz, $CDCl_3$) of compound 11

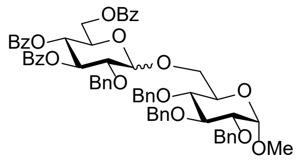

16

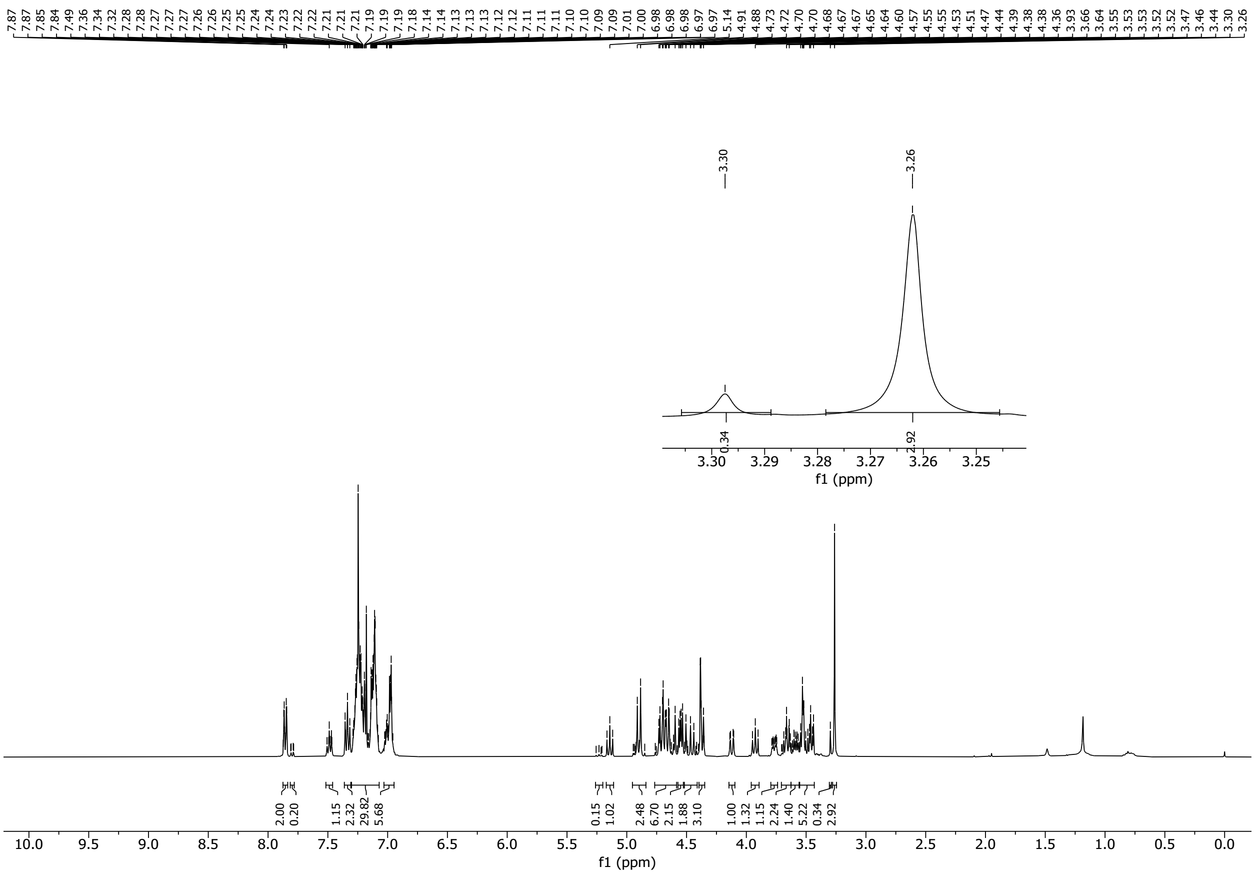

Figure S10. **^{1}H NMR spectrum (400 MHz, CDCl₃) of compound 16**

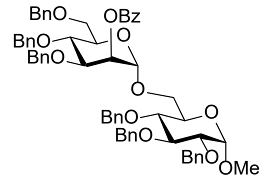

18

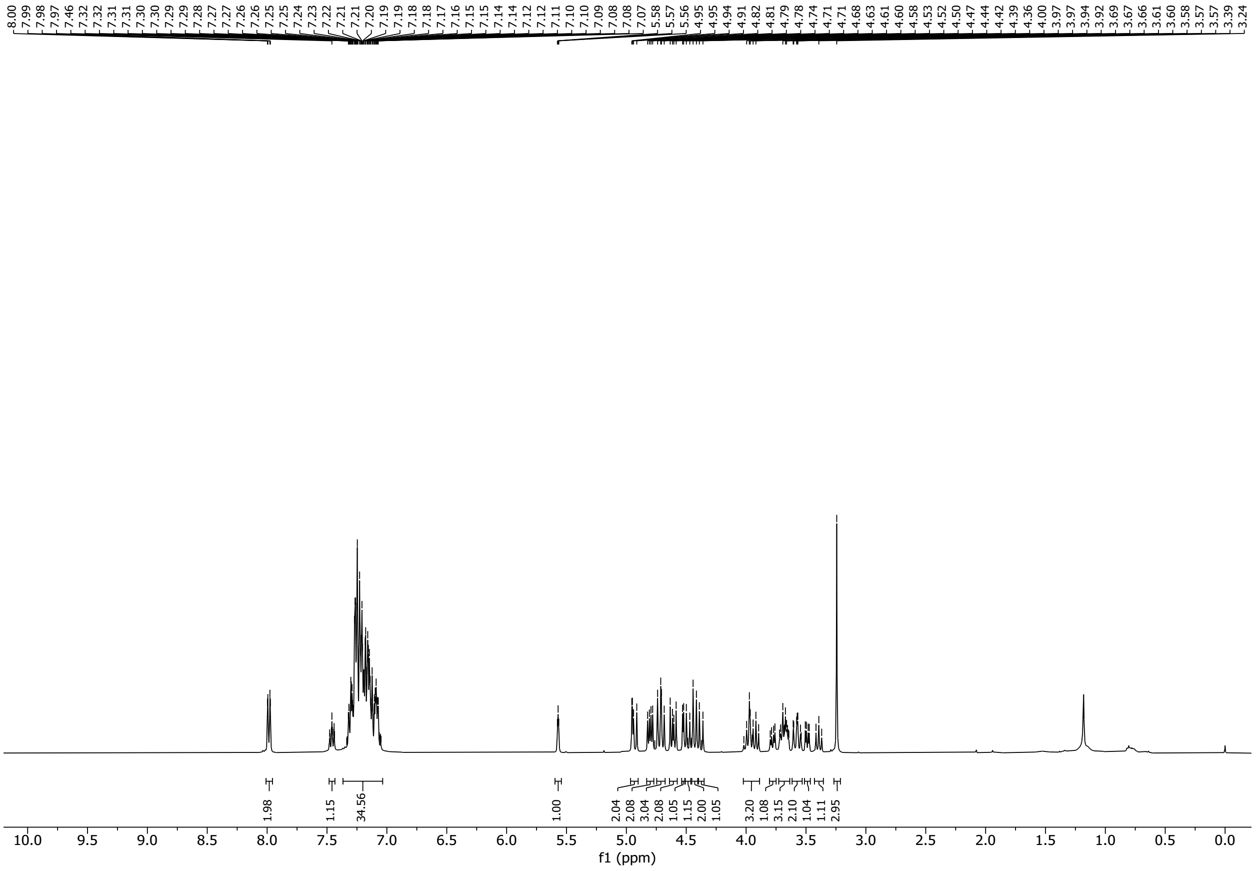

Figure S11. ¹H NMR spectrum (400 MHz, $CDCl_3$) of compound 18

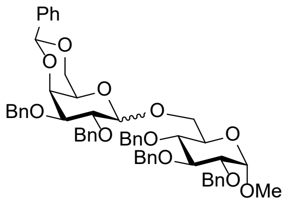

20

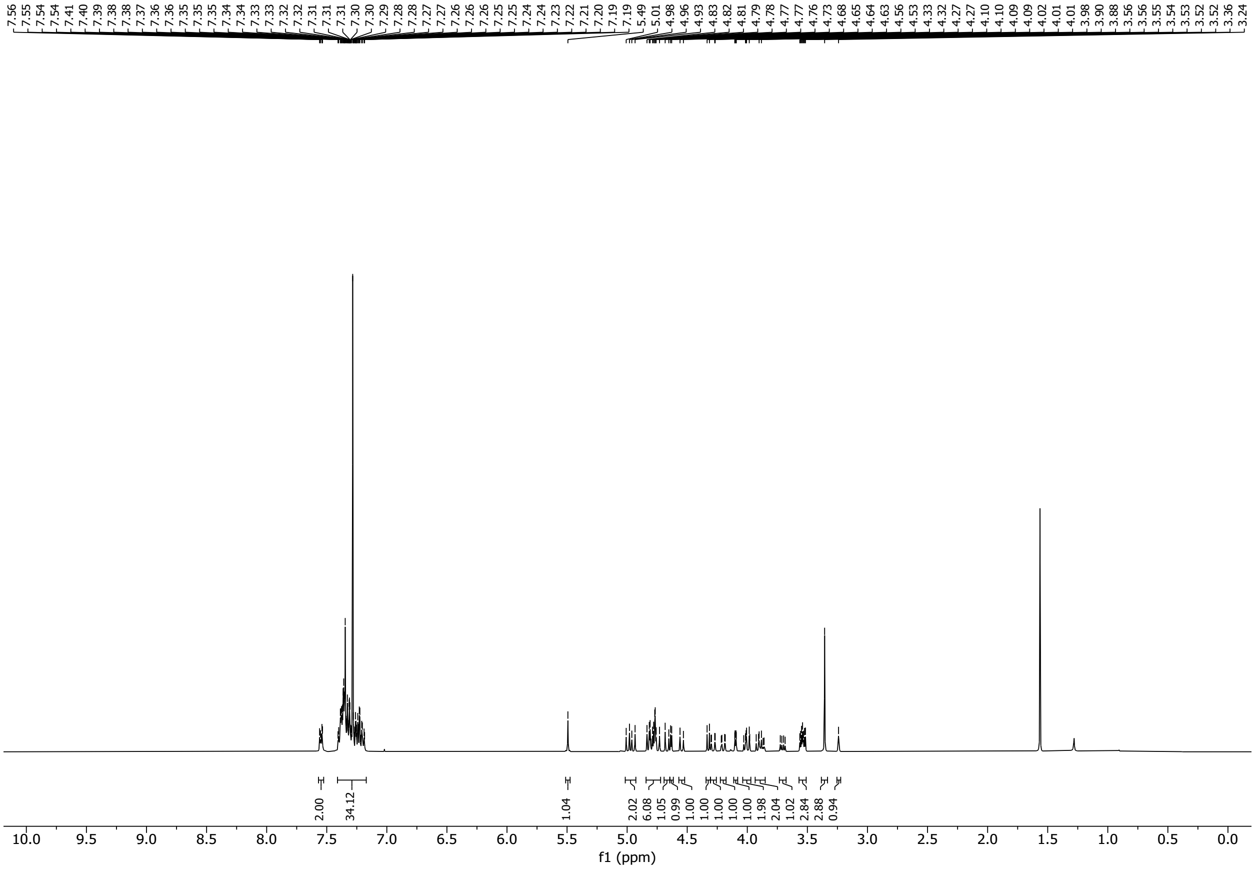

Figure S12. **^{1}H NMR spectrum (400 MHz, $CDCl_3$) of compound 20**

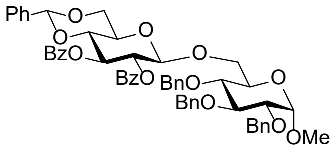

22

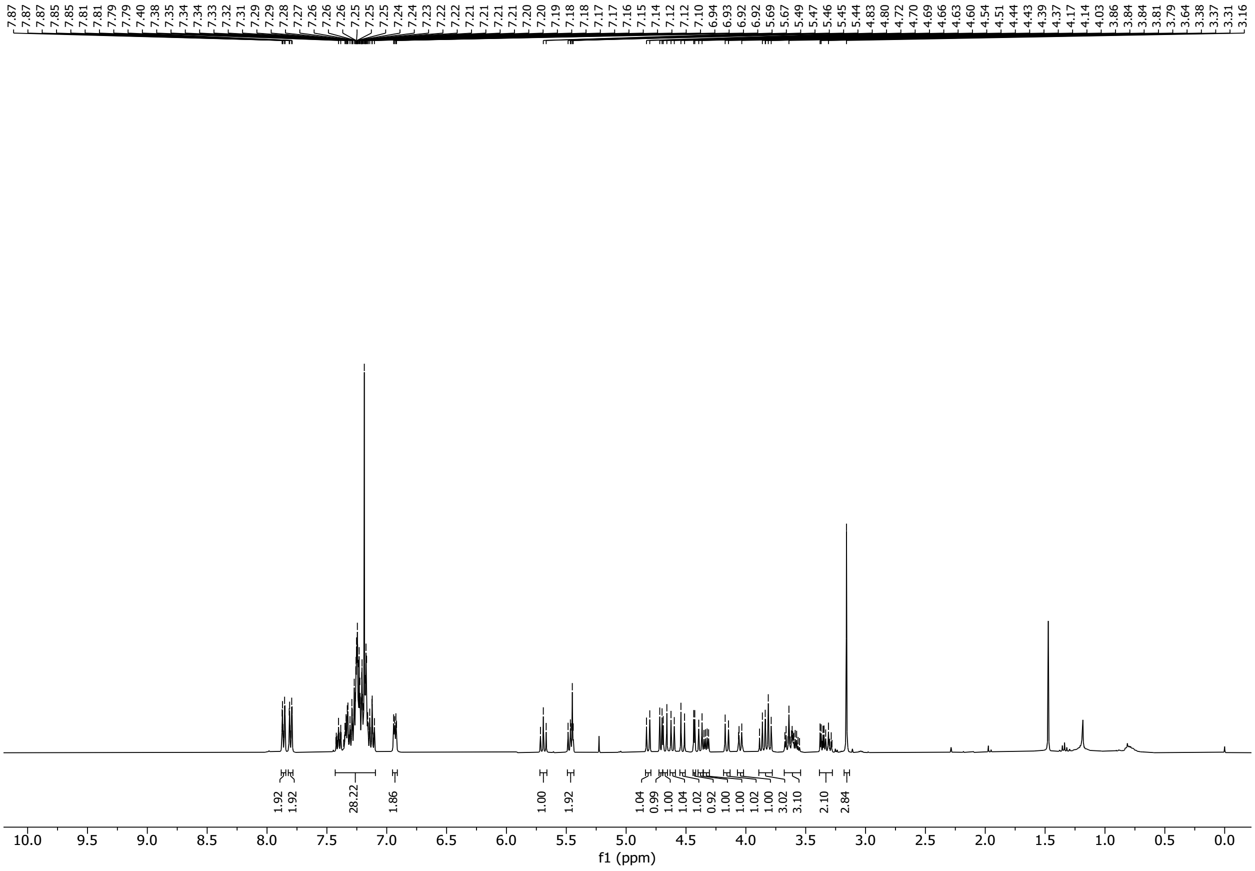

Figure S13. **^{1}H NMR spectrum (400 MHz, CDCl₃) of compound 22**

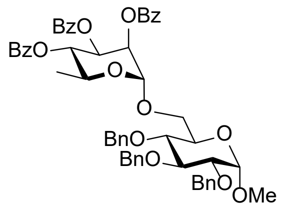

26

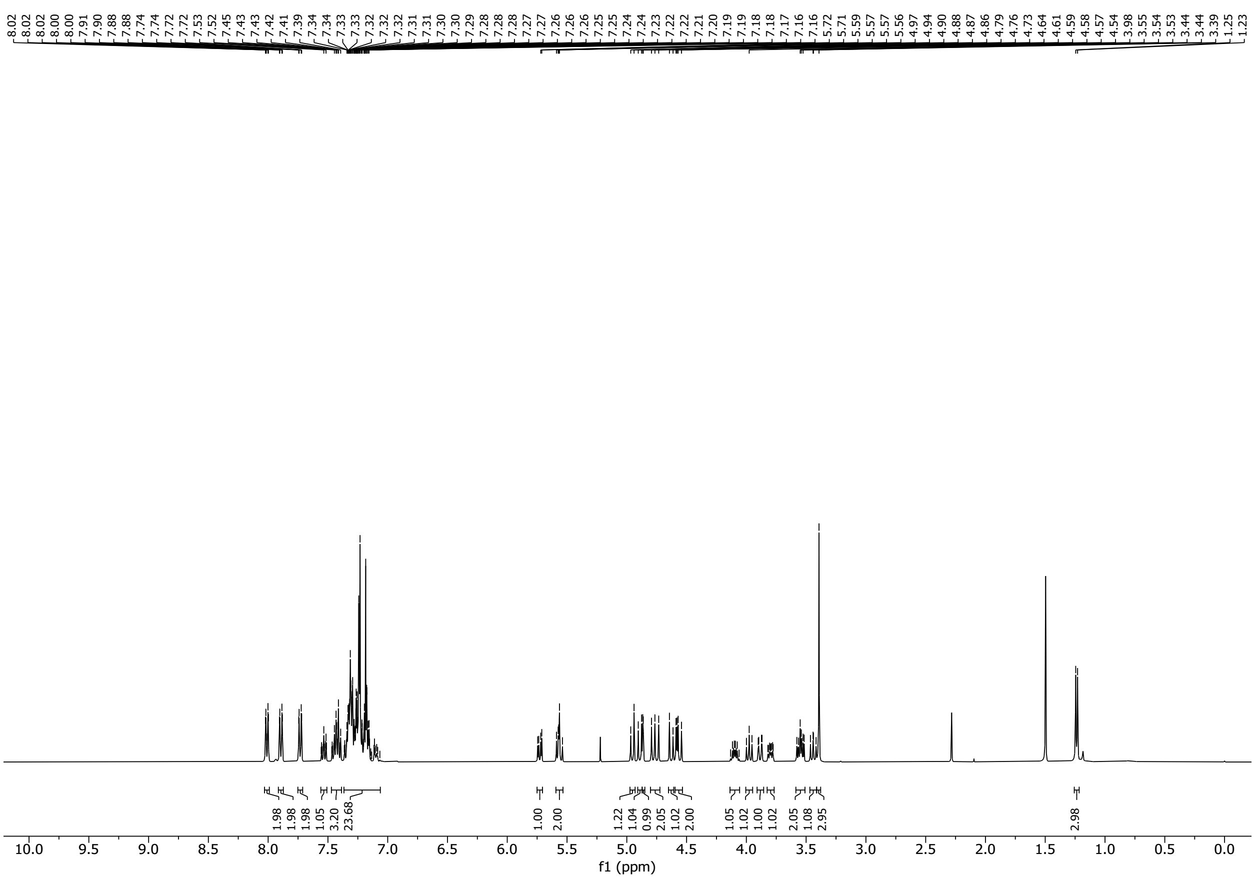

Figure S14. [1]**H NMR spectrum (400 MHz, CDCl**$_3$**) of compound 26**

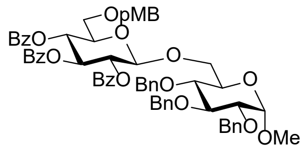

28

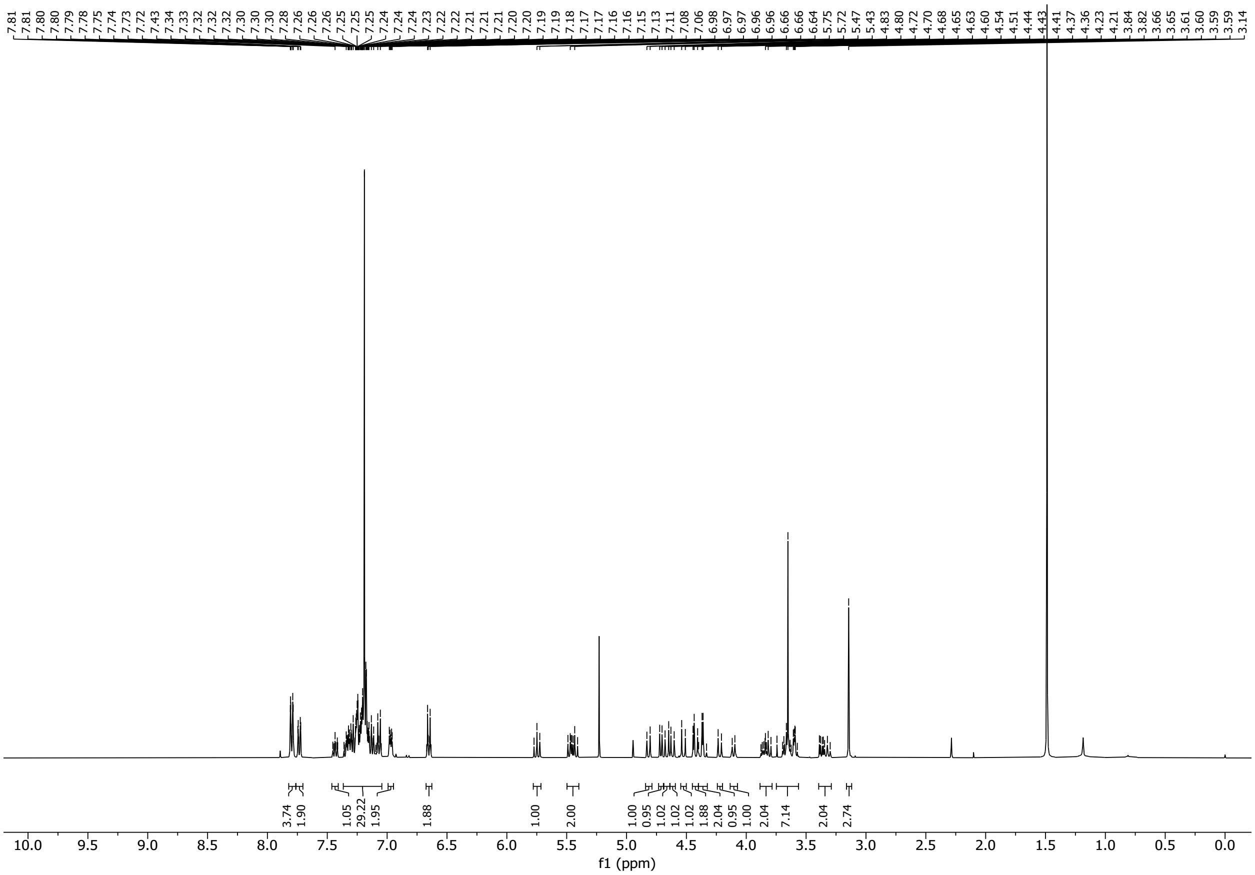

Figure S15. ^{1}H NMR spectrum (400 MHz, $CDCl_3$) of compound 28

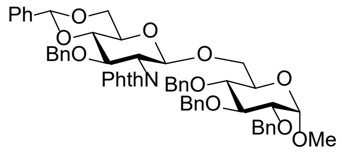

32

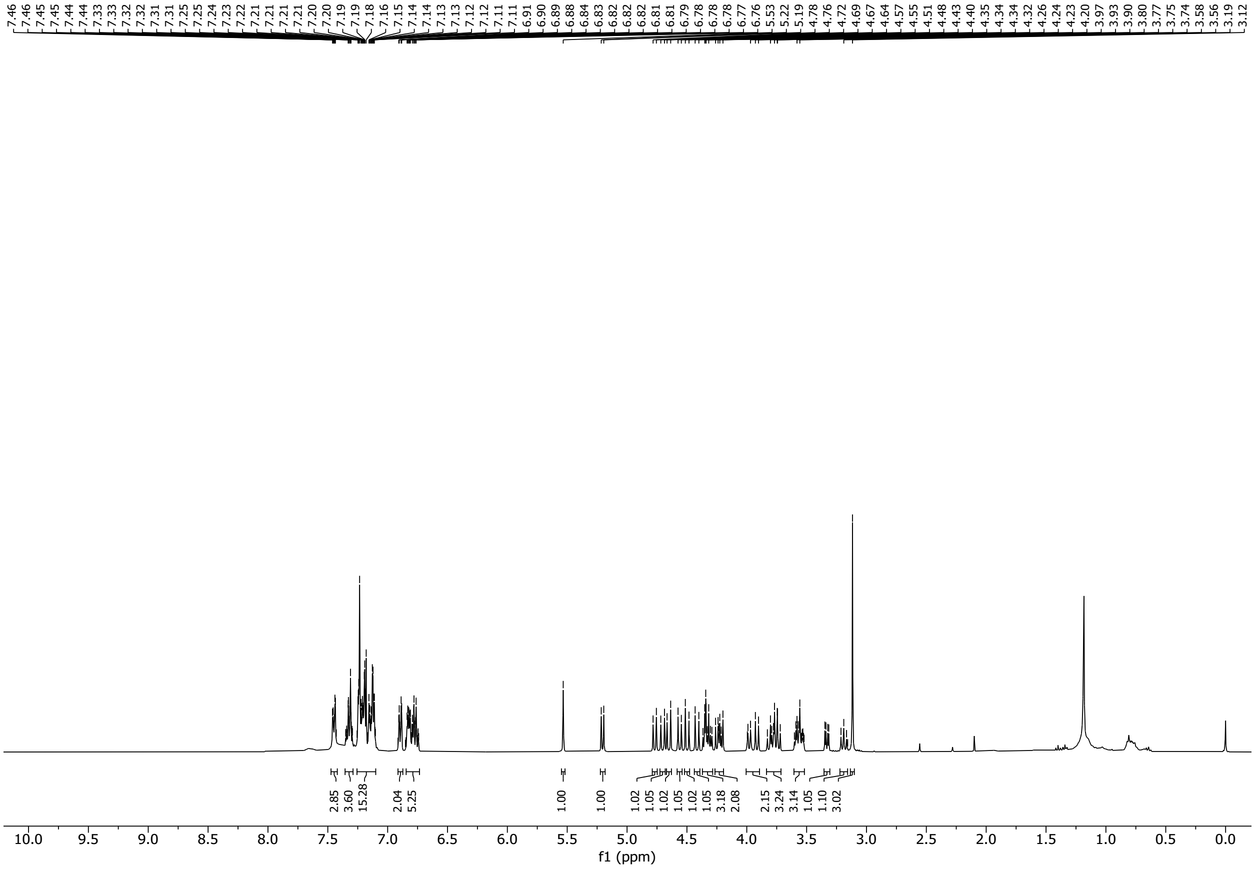

Figure S16. ^{1}H NMR spectrum (400 MHz, $CDCl_3$) of compound 32

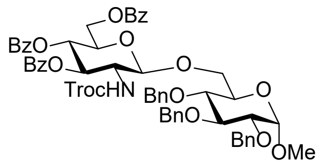

34

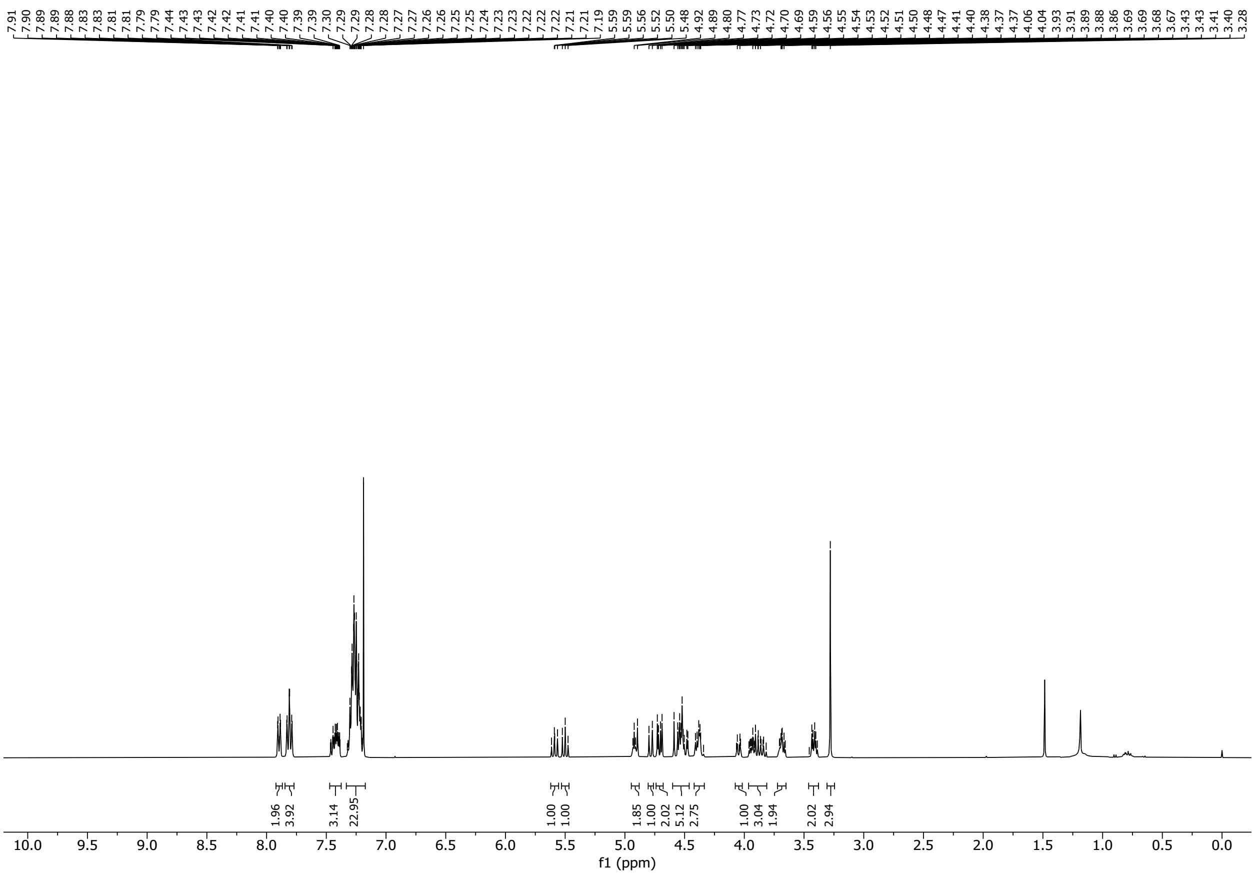

Figure S17. ^{1}H NMR spectrum (400 MHz, $CDCl_3$) of compound 34

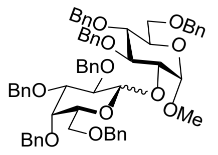

38

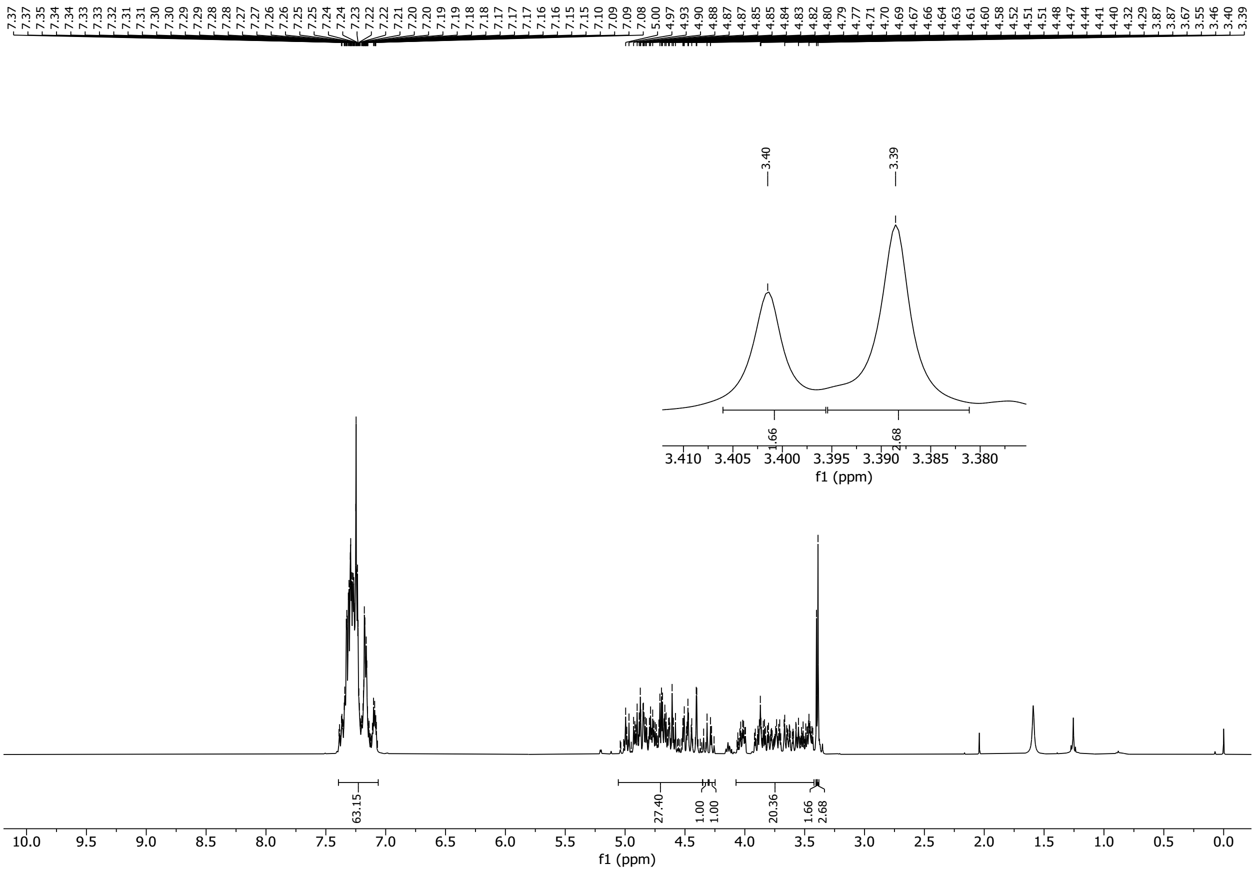

Figure S18. **^{1}H NMR spectrum (400 MHz, $CDCl_3$) of compound 38**

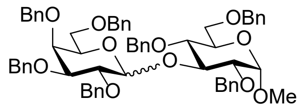

40

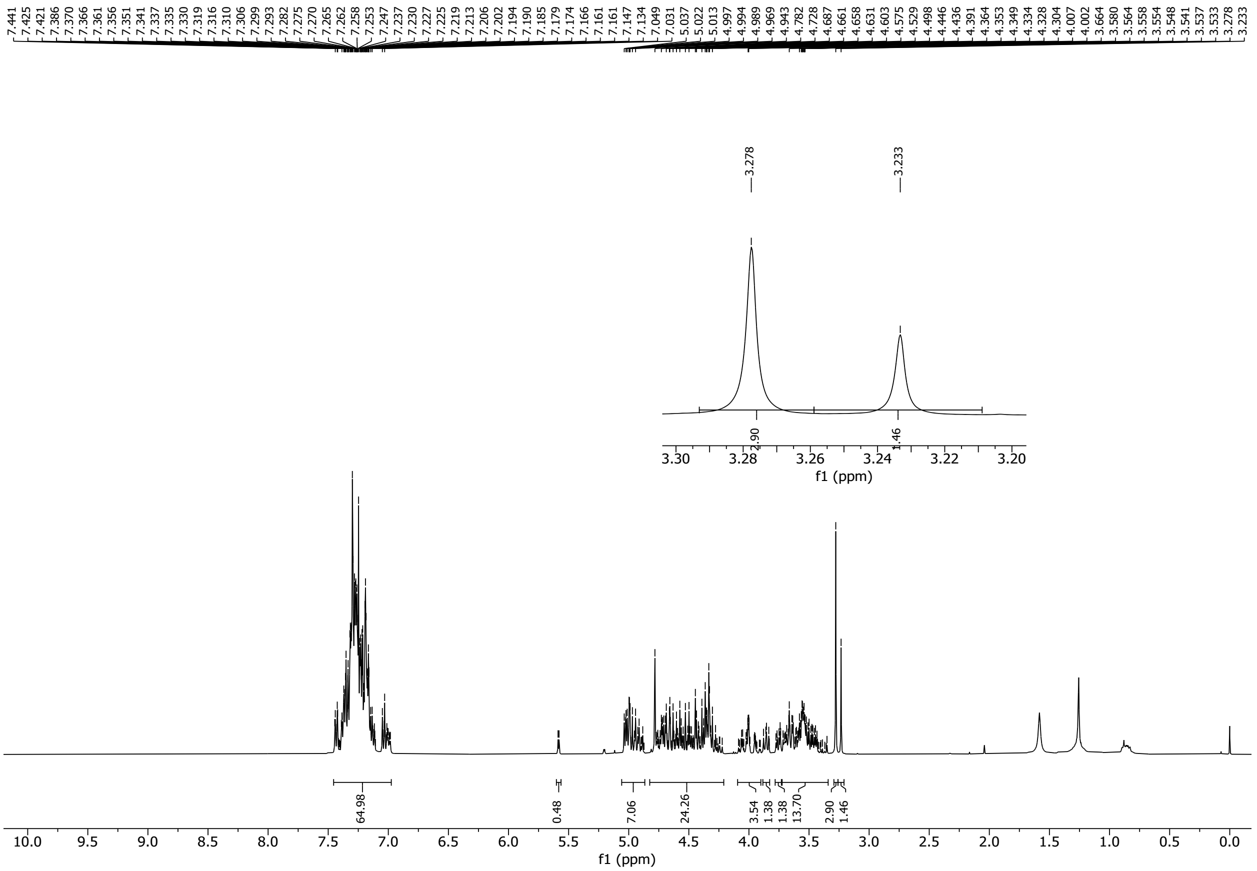

Figure S19. ^{1}H NMR spectrum (400 MHz, $CDCl_3$) of compound 40

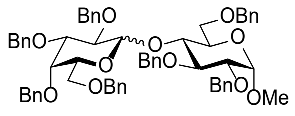

42

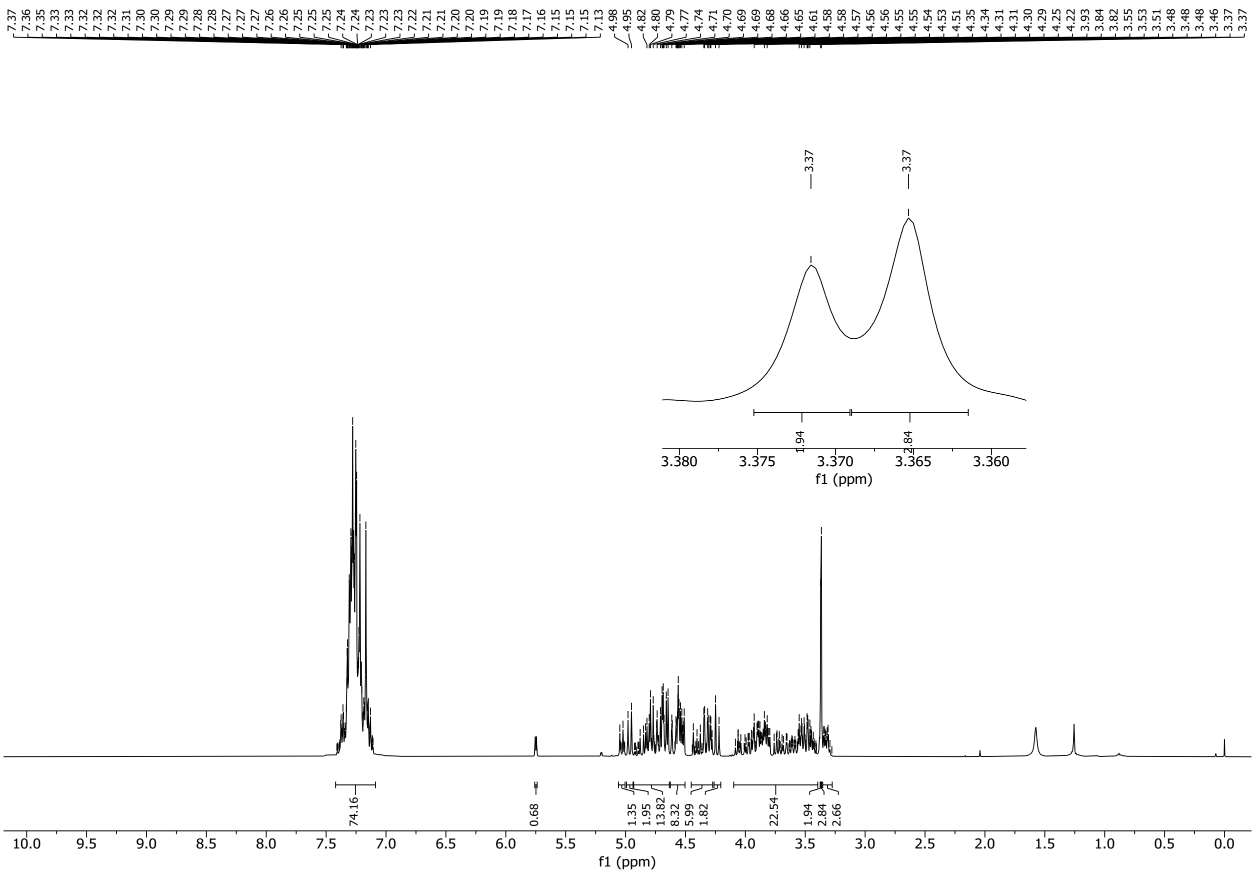

Figure S20. ^{1}H NMR spectrum (400 MHz, $CDCl_3$) of compound 42

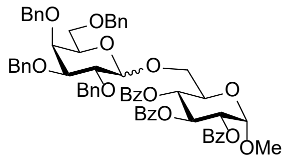

44

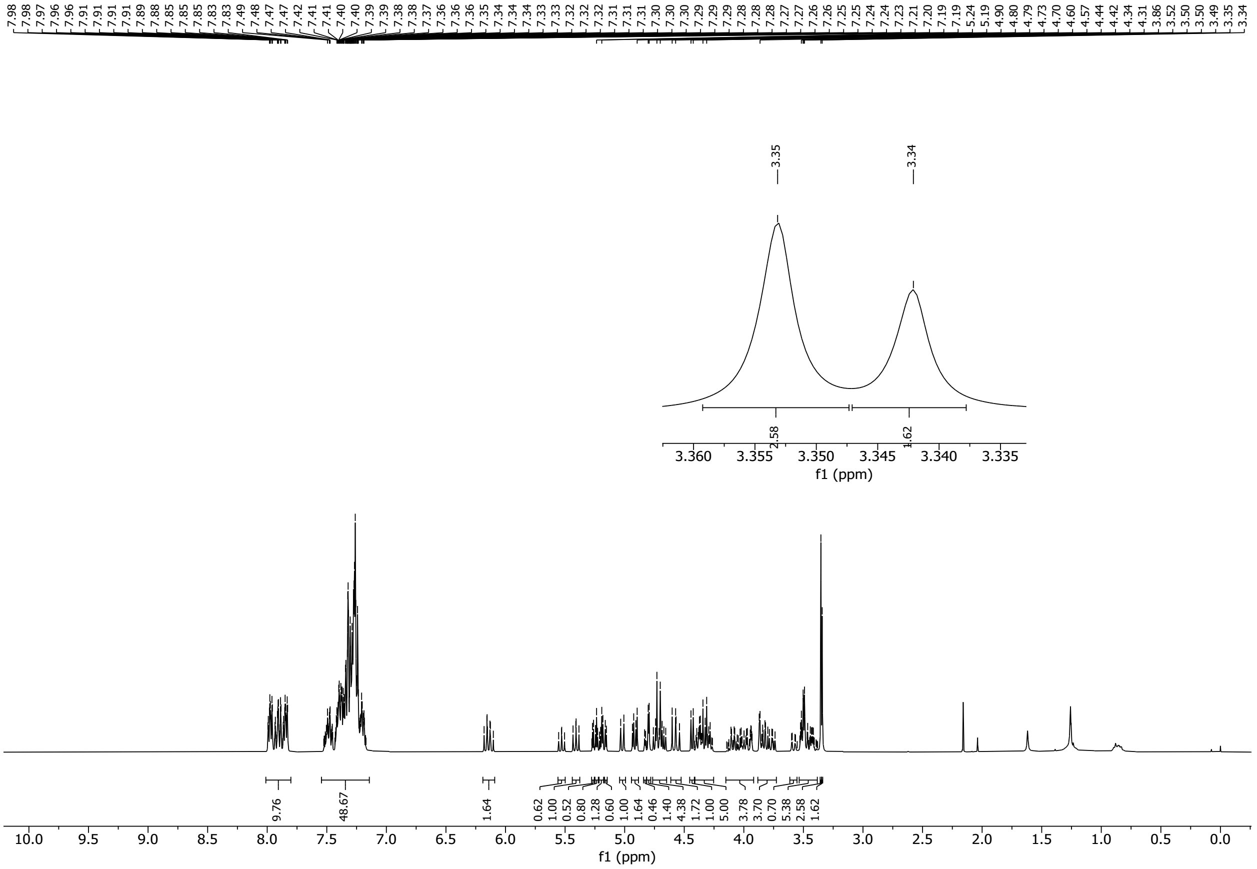

Figure S21. ^{1}H NMR spectrum (400 MHz, $CDCl_3$) of compound 44
